# Exosomes derived from bone marrow mesenchymal stem cells alleviate biliary ischemia reperfusion injury in fatty liver transplantation by inhibiting ferroptosis

**DOI:** 10.1007/s11010-023-04770-8

**Published:** 2023-05-27

**Authors:** Xuan Tian, Longlong Wu, Xiang Li, Weiping Zheng, Huaiwen Zuo, Hongli Song

**Affiliations:** 1 School of Medicine, Nankai University, Tianjin, People’s Republic of China; 2 Tianjin First Central Hospital Clinic Institute, Tianjin Medical University, Tianjin, 300070 People’s Republic of China; 3 Department of Organ Transplantation, Tianjin First Central Hospital, School of Medicine, Nankai University, No. 24 Fukang Road, Nankai District, Tianjin, 300192 People’s Republic of China; 4 NHC Key Laboratory of Critical Care Medicine, Tianjin, 300192 People’s Republic of China; 5 Tianjin Key Laboratory of Organ Transplantation, Tianjin, People’s Republic of China

**Keywords:** Exosome, Mesenchymal stem cell, Ferroptosis, Liver transplantation

## Abstract

Fatty liver grafts are susceptible to ischemia reperfusion injury (IRI), increasing the risk of biliary complications after liver transplantation (LT). Ferroptosis, a newly recognized programmed cell death, is expected to be a novel therapeutic target for IRI. We investigated whether exosomes derived from heme oxygenase 1-modified bone marrow mesenchymal stem cells (HExos) relieve ferroptosis and protect biliary tracts from IRI in a rat fatty liver transplantation model. Rats were fed with a methionine choline deficient (MCD) diet for 2 weeks to induce severe hepatic steatosis. Steatotic grafts were implanted and HExos were administered after liver transplantation. A series of functional assays and pathological analysis were performed to assess ferroptosis and biliary IRI. The HExos attenuated IRI following liver transplantation, as demonstrated by less ferroptosis, improved liver function, less Kupffer and T cell activation, and less long-term biliary fibrosis. MicroRNA (miR)-204-5p delivered by HExos negatively regulated ferroptosis by targeting a key pro-ferroptosis enzyme, ACSL4. Ferroptosis contributes to biliary IRI in fatty liver transplantation. HExos protect steatotic grafts by inhibiting ferroptosis, and may become a promising strategy to prevent biliary IRI and expand the donor pool.

**Supplementary Information:**

The online version contains supplementary material available at 10.1007/s11010-023-04770-8.

## Introduction

Organ shortage remains a major limitation encountered by liver transplantation centers worldwide. In the United States, approximately 20% of patients on waiting list have been reported to die due to organ shortage [1]. One strategy to alleviate the imbalance between supply and demand is to use extended criteria donor (ECD) livers, which are often discarded because of an increased risk of complications after implantation [2]. Due to the global prevalence of non-alcoholic fatty liver disease, moderate (macrosteatosis > 30%) or severe (macrosteatosis > 60%) steatotic liver is becoming an increasingly common type of ECD liver [3].

Steatotic grafts are more vulnerable to ischemia reperfusion injury (IRI) and are considered an important risk factor for post-transplant biliary complications, which affect the survival of recipients [4]. Ferroptosis, a novel type of regulated cell death characterized by iron-dependent accumulation of lipid peroxides, has been proven to be implicated in IRI [5]. Lipid peroxides eventually disrupt the biological membrane integrity, leading to cell death and the release of damage-associated molecular patterns (DAMPs), which recruit immune cells to initiate downstream aseptic inflammation.

Mesenchymal stem cell (MSC) therapy has become one of the most extensively studied cell therapies, and demonstrated promise in attenuating IRI and improving graft quality via a paracrine mechanism [6, 7]. MSC-derived exosomes contain a variety of cellular components, such as microRNAs (miRNAs), mRNAs, and proteins, mediate intercellular communication, and display similar functions with the parent MSCs [8, 9]. Compared to cell therapy, cell-free exosome therapy avoids the possible adverse effects associated with cell transplantation and improves safety for clinical application. MSC-derived exosomes have advantages over MSCs in terms of immune rejection and tumorigenicity [10]. Additionally, intravascular administration of exosomes presents a lower risk of vascular thrombosis [11]. Moreover, exosomes are more convenient to store and transport. The biological activity of exosomes is maintained at − 20 °C for 1 week and at − 80 °C for long-term storage [10].

Previously, we reported that heme oxygenase 1 (HO-1) genetic modification enhanced MSC survival and tissue repair abilities [12, 13]. In the present study, exosomes derived from HO-1-modified MSCs (HExos) were infused via the portal vein in fatty liver transplantation rat models. We found that the HExos improved the quality of steatotic grafts, relived IRI-induced inflammation, and attenuated long-term biliary fibrosis. Further analyses revealed that miR-204-5p delivered by the HExos negatively regulated ferroptosis by targeting the key pro-ferroptosis enzyme, ACSL4. The present study suggests that ferroptosis is a potential target for prevention and treatment of biliary IRI in fatty liver transplantation, and that HExos administration is a promising therapeutic strategy. These findings provide a novel perspective for improving the quality of steatotic grafts and expanding the donor pool.

## Materials and methods

### Animals

Male specific pathogen-free (SPF) Sprague–Dawley (SD) rats were obtained from China National Institutes for Food and Drug Control (Beijing, China) and reared in a standard laboratory setting. A total of 25 rats (40–80 g; 3–4 weeks old) were utilized for bone marrow MSC isolation, while a total of 96 rats (220–280 g; 7–8 weeks) were utilized to establish orthotopic liver transplantation (LT) models. All animal experiments were performed in accordance with current ethical guidelines, and were approved by the Animal Ethics Committee of Nankai University (Tianjin, China).

### Extraction and identification of MSCs

Extraction, culture, gene transfection and identification of MSCs were carried out as previously reported [12, 13]. In brief, MSCs were isolated from the bone marrow contents of femur and tibia using a continuous adherent method, and cultured in Dulbecco’s modified Eagle’s medium (DMEM)/F12 (Thermo Fisher Scientific, MA, USA) containing 10% fetal bovine serum (FBS; Biowest, Nuaille, France). MSCs were transfected with adenovirus (Jikai, Shanghai, China) to overexpress HO-1. HO-1-modified MSCs (HMSCs) were identified by in vitro osteogenic and adipogenic differentiation.

### Isolation and identification of exosomes

Exosomes were isolated by ultracentrifugation. Low-glucose DMEM containing 10% exosome-free FBS was used 48 h before exosome isolation. Then, the culture medium was collected and centrifuged at 4 ℃ and 10,000 g for 30 min to remove cell fragments. The supernatant was collected and centrifuged at 4 ℃ and 100,000 g for 70 min. The precipitations containing exosomes were resuspended with PBS and stored at − 80 ℃. Exosomes were characterized by transmission electron microscope HT7700 (Hitachi, Tokyo, Japan), and the particle size was determined by Nanosight NS300 (Malvern Instruments Ltd, Malvern, UK).

### Establishment of MCD fatty liver models

7–8 weeks SD rats were fed with methionine choline deficiency (MCD) diet (Medicience, Yangzhou, China) for 14 days. Fatty livers were obtained, and hematoxylin eosin (HE) staining and Oil Red O staining were performed.

### Liver transplantation models

Orthotopic liver transplantation (LT) was performed according to the two-cuff technique established by Kamada [14]. All operations were performed by the same doctor, and the anhepatic phase was 20 ± 3 min. Animals were randomly assigned to the following groups (*n* = 6): Control (Sham operation), LT (500 μL PBS/rat), MSC (2 × 10^6^ MSCs in 500 μL PBS/rat), HMSC (2 × 10^6^ HMSCs in 500 μL PBS/rat), Exo (2.5 × 10^10^ particles in 500 μL PBS/rat), and HExo (2.5 × 10^10^ particles in 500 μL PBS/rat). Cells or exosomes were injected through the portal vein immediately after LT. Animals were sacrificed 3 days after LT to collect samples for histology and liver functions. In addition, another Control group, LT group and HExo group (*n* = 6) were established and sacrificed 4 weeks after LT to detect long-term biliary injury. No animal was excluded from the analysis due to postoperative hemorrhage, infection, or death.

### *Tracking of exosomes *in vivo

Exosomes were incubated with CM-Dil dye (1 μM; Invitrogen, CA, USA) for 15 min, washed using PBS, and centrifuged at 100,000 g for 70 min. Then, the exosomes were washed using PBS again. LT was performed and the CM-Dil labeled exosomes (2.5 × 10^10^ particles/rat) were injected via the portal vein. Frozen sections of livers were obtained 6 h later and observed by fluorescence microscope.

### *Establishment of an *in vitro* model of IRI*

The IAR20 hepatic cell line was purchased from Procell Life Science & Technology Co., Ltd. (Wuhan, China). Human intrahepatic bile duct epithelial cells (HIBCs) were purchased from ICell Bioscience Inc. (Shanghai, China). The IRI cell model was established according to a previously published method [15]. Briefly, HIBCs or IAR20 cells were cultured in serum-free RPMI 1640 medium for 24 h, and then immersed in mineral oil to be deprived of oxygen and nutrients. After washing with PBS, cells were cultured in RPMI 1640 medium containing 10% FBS to mimic reperfusion.

### Cellular uptake of exosomes

Exosomes were labeled with CM-Dil dye as above. HIBCs were co-cultured with exosomes overnight and observed by confocal microscope FV1000 (Olympus, Tokyo, Japan).

### Cell viability

Cell Counting Kit-8 (CCK-8) (Solarbio, Beijing, China) was used to evaluate cell viability according to the manufacturer’s instructions. Optical density (OD) values were measured at 450 nm.

### Lipid peroxidation assay

Cells were dyed with BODIPY 581/591 C11 (5 μM; Thermo Fisher Scientific) for 20 min and washed in PBS. Lipid-reactive oxygen species (lipid-ROS) were examined by flow cytometry according to the manufacturer's instructions. Cells or tissues were homogenized and centrifuged to collect the supernatant. MDA assay kit (Beyotime, Shanghai, China) was used to detect in vivo and in vitro lipid peroxidation.

### Iron assay and glutathione assay

Cells or tissues were homogenized and centrifuged to collect the supernatant. Ferrous iron (Fe^2+^) level was determined by iron assay kit (Abcam, MA, USA). GSH assay kit (Solarbio, Beijing, China) was used for glutathione (GSH) detection.

### Western blot

Tissues or cells were lysed by radioimmunoprecipitation assay lysis buffer (Solarbio). The proteins were separated by SDS-PAGE and transferred to PVDF membranes. The membranes were incubated overnight using the following primary antibodies: ACSL4 (Santa Cruz, Dallas, USA), GPX4 (Santa Cruz), HMGB1 (Proteintech, Wuhan, China), HO-1 (Proteintech), α-SMA (Proteintech), GAPDH (Beyotime) and β-actin (Beyotime). The membranes were exposed using a ChemiDoc XRS + system (Bio-Rad, Hercules, CA, USA).

### Extraction of RNA, qRT-PCR, and RNA-seq

Trizol (Takara, Shiga, Japan) was used to extract total RNA. Reverse transcription and qRT-PCR were performed using SYBR Green PCR Master Mix (Takara) according to the manufacturer’s instructions. β-actin was used for mRNA standardization and U6 for miRNA standardization. The primer sequences are shown in S1. RNA sequencing (RNA-seq) and data analysis were performed by BGI Genomics (Shenzhen, China). The RNA-seq data were deposited in Sequence Read Archive (PRJNA795289). Differentially expressed genes were used for heat map analysis and KEGG enrichment analysis. *P* < 0.05 was considered as significant enrichment.

### Histology and IF

Liver tissues were fixed in 4% formalin and embedded in paraffin to obtain 4 μm sections. Liver sections were stained with hematoxylin and eosin (HE), and liver histological damage was evaluated with the Suzuki score [16]. For immunohistology (IHC), paraffin sections were deparaffinized, blocked with 5% normal goal serum, and incubated overnight with the following antibodies: ACSL4 (Santa Cruz), HMGB1 (Proteintech), CD68 (Abcam), 4-HNE (Abcam). Masson staining kit (Solarbio) was used to detect collagenous connective tissue fibers. For immunofluorescence (IF), cells were fixed with 4% paraformaldehyde and paraffin sections were deparaffinized. Cells or sections were incubated with the following antibodies: ACSL4 (Santa Cruz), HMGB1 (Proteintech), CD3 (Abcam). All slides were observed under an ECLIPSE Ni microscope (Nikon, Tokyo, Japan) and images (96-dpi resolution) were obtained under × 40 or × 100 magnification by NIS-Elements software (Nikon).

### Biochemical examination

Serum alanine aminotransferase (ALT), aspartate aminotransferase (AST) and total bilirubin (TBIL) were detected by biochemical analyzer Cobas 800 (Roche, Switzerland).

### Transmission electron microscope

Liver tissues were fixed with 2.5% glutaraldehyde, dehydrated in gradient ethanol, embedded in epoxy resin, cut into 100 nm ultra-thin sections, stained with uranyl acetate and lead citrate, and observed by transmission electron microscope HT7700 (Hitachi).

### Prediction of miRNA-mRNA targeting relationship

MiRDB [17], TargetScan [18], and miRWalk [19] were used to predict the mRNA targets of relevant miRNAs.

### Dual‑luciferase reporter assay

Wild-type (WT) and mutated (MUT) forms of the 3′-UTR of *ACSL4* were cloned to construct WT and MUT plasmids (Shitaike, Tianjin, China). HEK 293 T cells were co-transfected with the luciferase reporter plasmid and NC-mimic or miR-204-5p-mimic. The activity of firefly luciferase and Renilla luciferase was estimated.

### Transient transfection

Lipofectamine 3000 (Invitrogen) was used to transfect miR-204-5p mimics and miR204-5p inhibitor. The sequences of miR-204-5p mimics and miR204-5p inhibitor are shown in Table S2.

### Flow cytometry

After erythrocyte lysis, peripheral blood samples were incubated with the following fluorescent antibodies: CD3 (BioLegend, CA, US), CD4 (BioLegend), and CD8 (BioLegend). Fluorescence was detected by FACS Canto II (BD Biosciences, CA, USA). Mitochondrial membrane potential was measured using JC-1 kits (Beyotime) according to the manufacturer's instructions. Annexin V/PI kit (Solarbio) was used for cell death analysis.

### Statistical analysis

The data were analyzed by one-way ANOVA or Student’s *t* test and presented as mean ± SD. Normality was tested via the Shapiro–Wilk test. All data were analyzed using GraphPad Prism 9 (GraphPad Software, San Diego, CA). *P* < 0.05 was considered to be statistically significant.

## Results

### Hexos attenuate IRI of fatty liver grafts

**Fig. 1:**
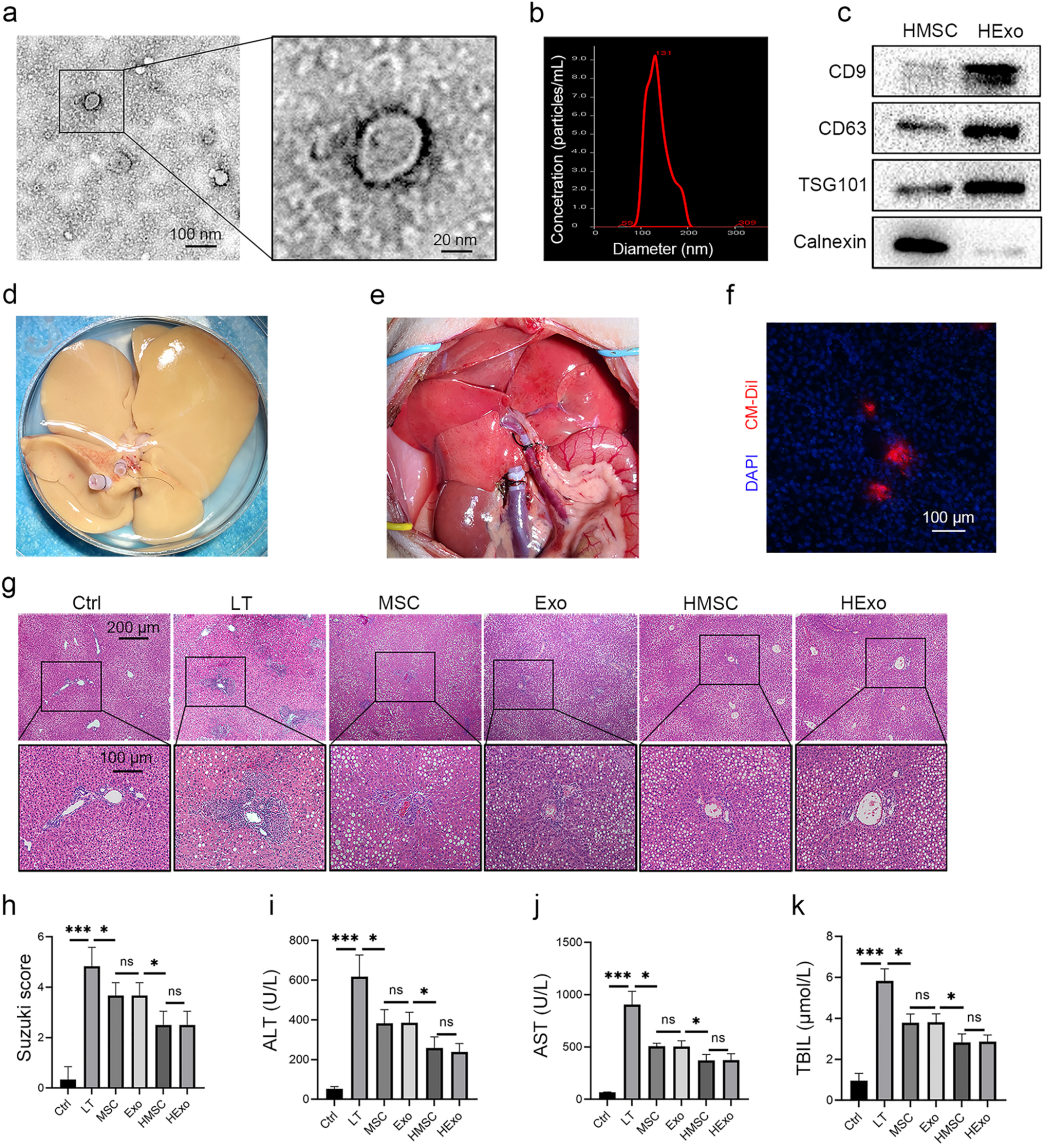
HExos attenuate IRI in fatty liver transplantation models. **a** Observation of HExos under transmission electron microscope. **b** NTA analysis of HExos. **c** Detection of markers on the surface of HExos. **d** Steatotic graft procurement. **e** Steatotic graft was implanted, and HExos were injected through the portal vein. **f** Frozen sections showing the presence of CM-Dil-labeled HExos (red fluorescence) in the liver. **g** Representative HE staining images of livers 3 days after LT. **h** Suzuki score was used to evaluate hepatic IRI severity. **I**–**k** Liver functions were detected 3 days after LT (*n* = 6). *ALT* alanine aminotransaminase, *AST* aspartate transaminase, *Exo* exosome, *NTA* Nanoparticle tracking analysis, *HExo* exosome derived from HO-1-modified mesenchymal stem cells, *HMSC* HO-1-modified mesenchymal stem cells, *IRI* ischemia reperfusion injury, *LT* liver transplantation, *MSC* mesenchymal stem cell, *TBIL* total bilirubin

Bone marrow MSCs were isolated and transfected with HO-1 (Fig. S1). Exosomes were extracted from the supernatant of HO-1-modified MSCs (HMSCs), and characterized by circular bilayer structure with particle sizes ranging from 50 to 150 nm (Fig. 1a, b). Compared to HMSCs lysis, exosomes derived from HMSCs (HExos) were positive for the exosome-related markers CD9, CD63 and TSG101, and negative for Calnexin (Fig. 1c).

Fatty liver rat models were established via the MCD diet (Fig. S2), and utilized for transplantation. The fatty liver graft had a yellowish and uniform appearance after procurement, and turned pink rapidly once implanted (Fig. 1d, e). To clarify the biodistribution of exosomes in the liver, CM-Dil labeled HExos (2.5 × 10^10^ particles/100 μL) were injected through the portal vein, and frozen sections demonstrated that the HExos were localized in the liver 6 h after injection (Fig. 1f).

PBS, MSCs, exosomes derived from MSCs (Exos), HMSCs, or HExos were injected via the portal vein after LT. Histological analysis showed significant IRI at 3 days postoperatively, manifested as hepatic sinusoidal dilatation and congestion, massive inflammatory cell infiltration, biliary epithelium exfoliation, and bile duct atresia (Fig. 1g). The administration of MSCs or Exos attenuated histological injury and reduced Suzuki score for liver injury, and the HMSCs or HExos showed better therapeutic efficacy (Fig. 1g, h). Furthermore, the HMSCs or HExos improved liver functions much more than the MSCs or Exos (Fig. 1i–k). The exosomes demonstrated similar therapeutic efficacy to their parent cells.

### Ferroptosis is implicated in IRI

**Fig. 2:**
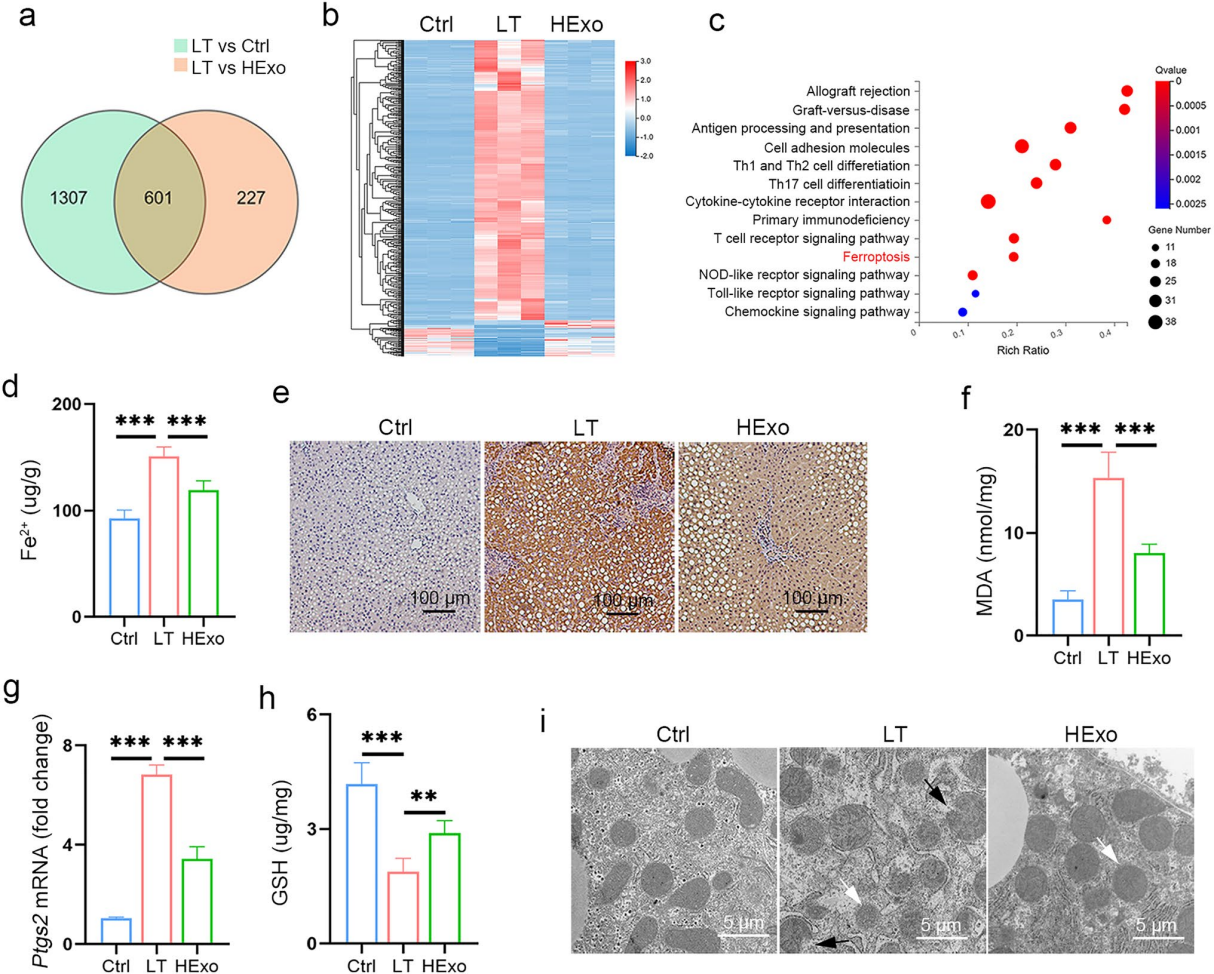
Ferroptosis is implicated in IRI. RNA-seq was performed in the Ctrl, LT, and HExo groups. **a** Venn diagram showing 601 DEGs. **b** Heatmap showing the DEG profile based on the 541 up-regulated and 60 down-regulated genes. **c** KEGG enrichment analysis of the 601 DEGs. **d** Fe^2+^ level was measured by iron assay kit. **e** 4-HNE was detected by immunohistochemistry. **f**–**h** MDA (f), *Ptgs2* mRNA (**g**), and GSH (**h**) levels were measured (*n* = 6 mice per group). i Representative TEM images of livers. White arrow indicates diminished or absent mitochondria cristae, and black arrow indicates mitochondrial membrane rupture. Data are presented as the means ± standard deviation. *P < 0.05, **P < 0.01, ***P < 0.001. *DEG* differentially expressed gene, *GSH* glutathione, *HExo* exosome derived from HO-1-modified mesenchymal stem cells, *LT* liver transplantation, *MDA* malondialdehyde

In order to investigate the underlying mechanisms of HExos alleviation of IRI, RNA-seq was performed in the Ctrl, LT and HExo groups, and 601 differentially expressed genes (DEGs) were identified (Fig. 2a). Heat map showed 541 upregulated and 60 downregulated genes (fold change > 2, *P* < 0.05) (Fig. 2b, Table S3). KEGG pathway enrichment analysis demonstrated that the DEGs were mainly enriched in pathways associated with transplantation immunology, such as allograft rejection, graft-versus-host disease, and antigen presentation (Fig. 2c). Notably, ferroptosis was one of the top 10 enriched KEGG pathways (Fig. 2c). Recent studies demonstrated that ferroptosis was important in the IRI pathophysiological processes. Therefore, we measured ferroptosis in fatty LT and assessed the therapeutic efficacy of HExos.

LT induced a robust increase in Fe^2+^ levels and the molecular markers of ferroptosis, such as *Ptgs2* mRNA, 4-hydroxynonenal (4-HNE), and malondialdehyde (MDA) (Fig. 2d–g). Glutathione (GSH), an important antioxidant maintaining cellular redox homeostasis, decreased after LT (Fig. 2h). The mitochondria of the LT group exhibited typical ferroptotic characteristics, such as diminished or complete absence of mitochondria cristae and outer membrane rupture (Fig. 2i). These results revealed that ferroptosis is closely implicated in IRI. The administration of HExos reduced the elevated level of Fe^2+^ and the molecular markers of ferroptosis induced by LT, and increased GSH levels (Fig. 2d–h). Furthermore, HExos improved mitochondrial status (Fig. 2i).

**Fig. 3:**
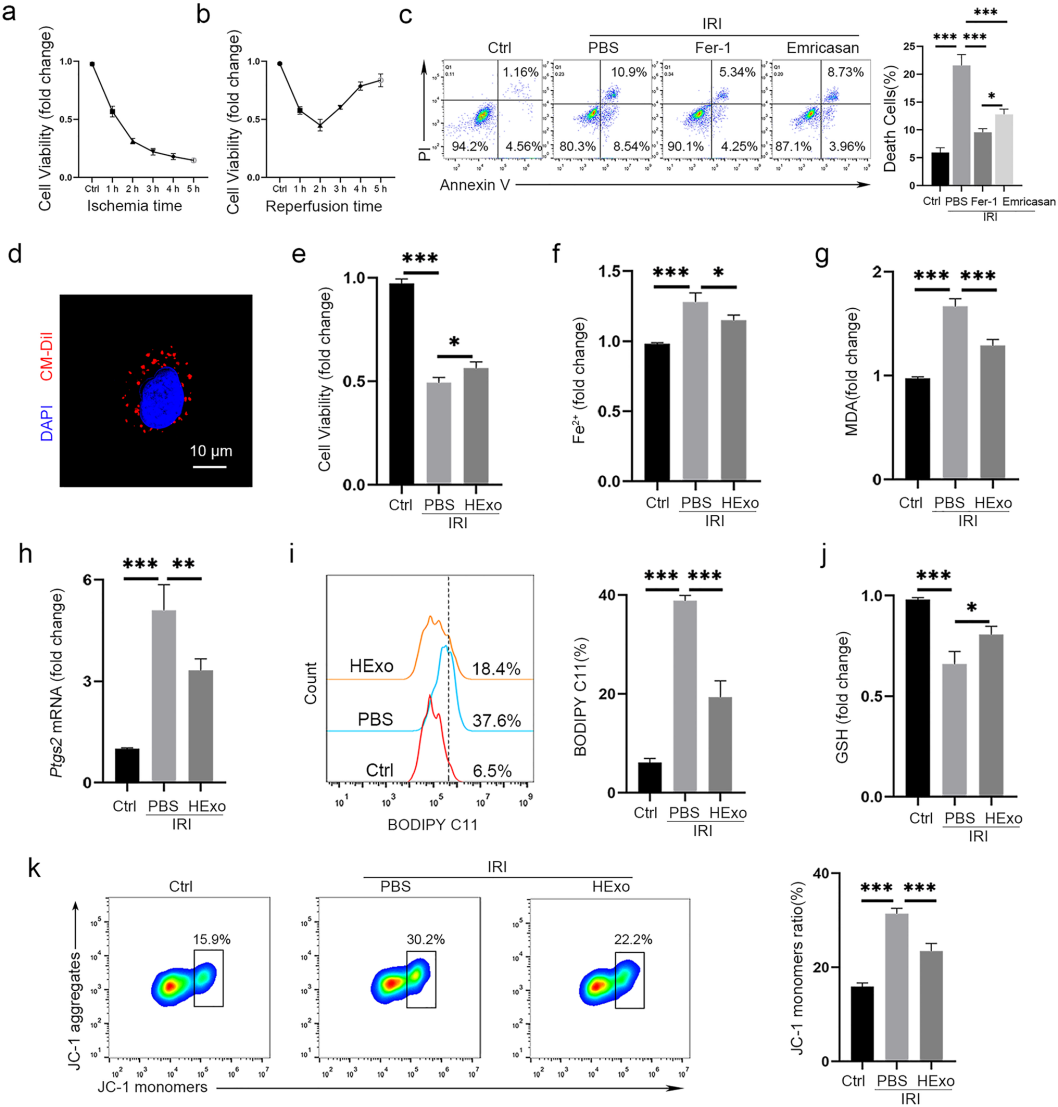
HExos attenuate IRI by inhibiting ferroptosis. **a** HIBCs were immersed in mineral oil to mimic ischemia, and cell viability was measured by CCK-8 assay. **b** HIBCs were immersed in mineral oil for 1 h, then cultured in complete medium to mimic reperfusion. Cell viability was measured by CCK-8 assay. **c** In vitro model of 1-h ischemia and 2-h reperfusion was established, and ferrostatin-1 (1 μM) or emricasan (20 μM) was administered. Annexin V/PI staining was performed and the proportion of PI positive cells was used as an indicator of cell death rate. **d** CM-Dil-labeled HExos (red fluorescence) were taken up by HIBCs. **e**–**k** HExos were administered in the in vitro model of IRI, and cell viability (**e**) and the levels of Fe^2+^ (**f**), MDA (**g**), *Ptgs2* mRNA (**h**), lipid-ROS (**i**), and GSH (**j**) were detected. **k** Mitochondrial membrane potentials were detected by JC-1 kit. Data are presented as the means ± standard deviation. The experiments were repeated three times. *P < 0.05, ***P < 0.001. *GSH* glutathione, *HExo* exosome derived from HO-1-modified mesenchymal stem cells, *MDA* malondialdehyde

An in vitro IRI model was established by mineral oil immersion. The CCK-8 assay revealed that HIBC viability was lowest after hypoxia for 1 h and reoxygenation for 2 h (Fig. 3a, b). Under identical conditions, the HIBCs were more susceptible to hypoxia than hepatic cells (Fig. S3), which partly explained the high incidence of biliary complications after LT. In order to explore relative contributions of different forms of cell death to biliary IRI, cell viability was measured in the presence of different small molecules. Ferrostatin-1, a ferroptosis inhibitor, was more effective than emricasan (an apoptosis inhibitor) for reducing IRI-induced cell death (Fig. 3c).

HExos were administered in the in vitro IRI model. To demonstrate the cellular uptake of exosomes, CM-Dil labeled HExos (2.5 × 10^9^ particles) were co-cultured with HIBCs for 12 h. Confocal microscopy revealed that the HExos were taken up by the HIBCs (Fig. 3d). Consistent with the in vivo studies, the HExos effectively reduced cell death, Fe^2+^ levels, and the molecular markers of ferroptosis (MDA, *Ptgs*2 mRNA, and lipid-ROS), and GSH levels were increased (Fig. 3e–j). Additionally, the mitochondrial membrane potential assay demonstrated that HExos rescued mitochondrial damage (Fig. 3k).

### Exosomes derived from HMSCs alleviate ferroptosis induced by LT

**Fig. 4:**
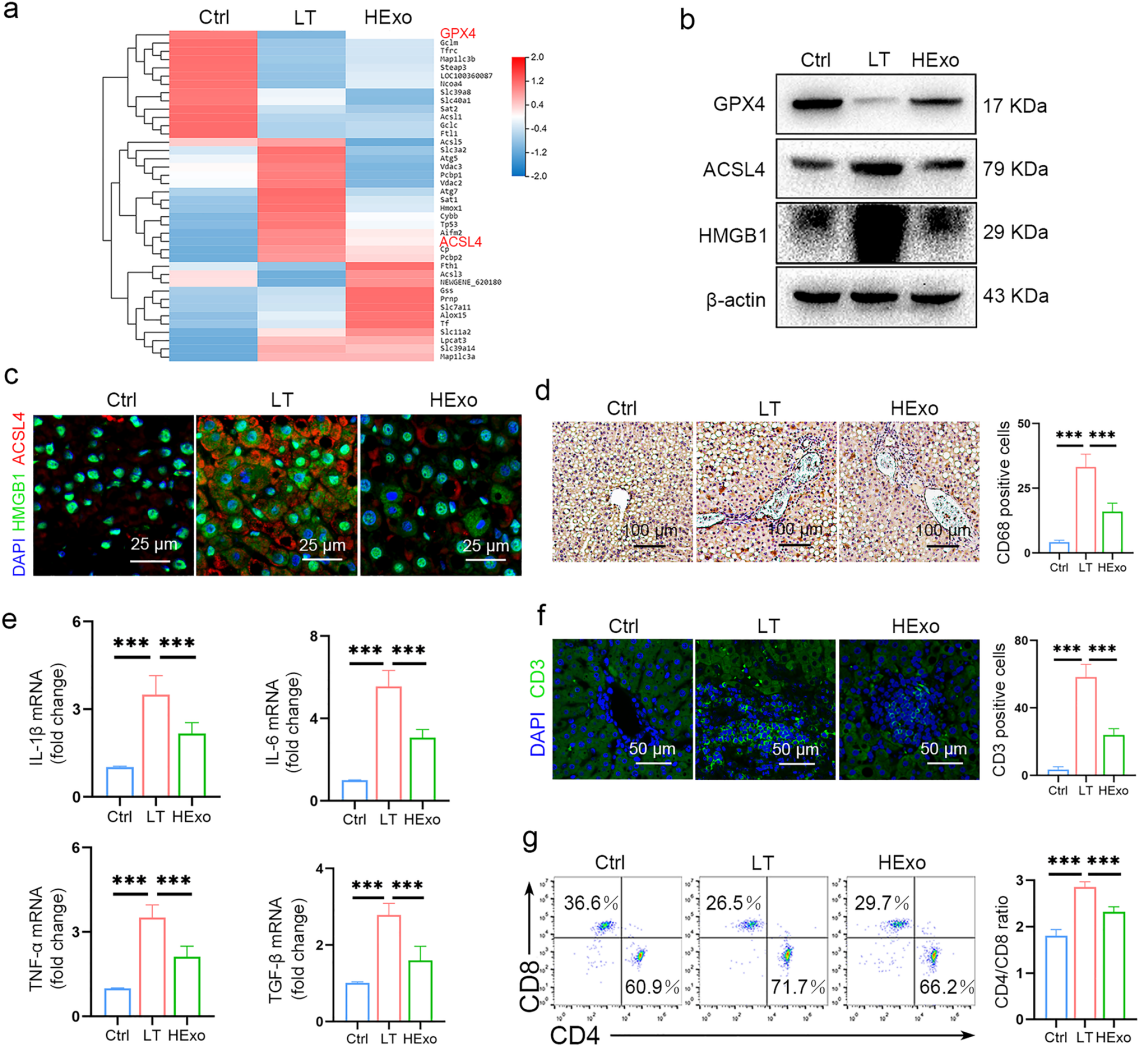
HExos attenuate ferroptosis and the downstream inflammation. **a** Heatmap showing the ferroptosis-related genes of RNA-seq. **b** Protein levels of GPX4, ACSL4, and HMGB1. **c** Expression level and subcellular localization of HMGB1 (green) and ACSL4 (red). **d** CD68-positive Kupffer cells were detected by immunohistochemistry. **e** mRNA levels of the inflammatory cytokines, including IL-1β, IL-6, TNF-α, and TGF-β, were detected by qRT-PCR (*n* = 6 mice per group). **f** T cells recruitment in the liver. **g** CD4/CD8 ratio in peripheral blood. Data are presented as the means ± standard deviation. *P < 0.05, **P < 0.01, ***P < 0.001. *HExo* exosome derived from HO-1-modified mesenchymal stem cells, *LT* liver transplantation, *TEM* transmission electron microscope

Given the important role of ferroptosis in IRI, we conducted further analysis of ferroptosis-related genes and determined that pro-ferroptosis genes were upregulated in the LT group, while anti-ferroptosis genes were downregulated (Fig. 4a, Table S4). The HExos rescued this trend somewhat, which suggested that the HExos relieved IRI by inhibiting ferroptosis. ACSL4 is a key regulator of ferroptosis, and RNA-seq demonstrated that Hexos administration reduced the increased ACSL4 level induced by LT, which was confirmed by western blot (Fig. 4a, b). Meanwhile, GPX4, a critical protective enzyme that prevents ferroptosis, was downregulated in the LT group and rescued by HExos (Fig. 4a, b).

HMGB1 is an endogenous DAMP released from the nucleus into the cytoplasm to activate immune cells and trigger inflammation when cells were damaged or dead. HMGB1 was significantly upregulated and translocated to the cytoplasm in the LT group and downregulated after HExos administration (Fig. 4b, c).

Kupffer cells are activated by HMGB1 [20] and secrete a considerable range of cytokines during hepatic IRI, orchestrating inflammation and fibrosis [21]. The LT group had significantly more CD68-positive Kupffer cells (Fig. 4d), and had elevated levels of proinflammatory cytokines (IL-1β and IL-6) and pro-fibrotic cytokines (TNF-α and TGF-β) (Fig. 4e). Large numbers of T cells were recruited to the liver, and the CD4 + /CD8 + T cell ratio increased (Fig. 4f, g). The CD4/CD8 ratio is an immune indicator with a reference value in the range 1.4–2.0, which increases when transplantation rejection occurs. HExos effectively alleviated inflammatory response induced by HMGB1 (Fig. 4d–g).

### HExos alleviate ferroptosis by delivering miR-204-5p

**Fig. 5:**
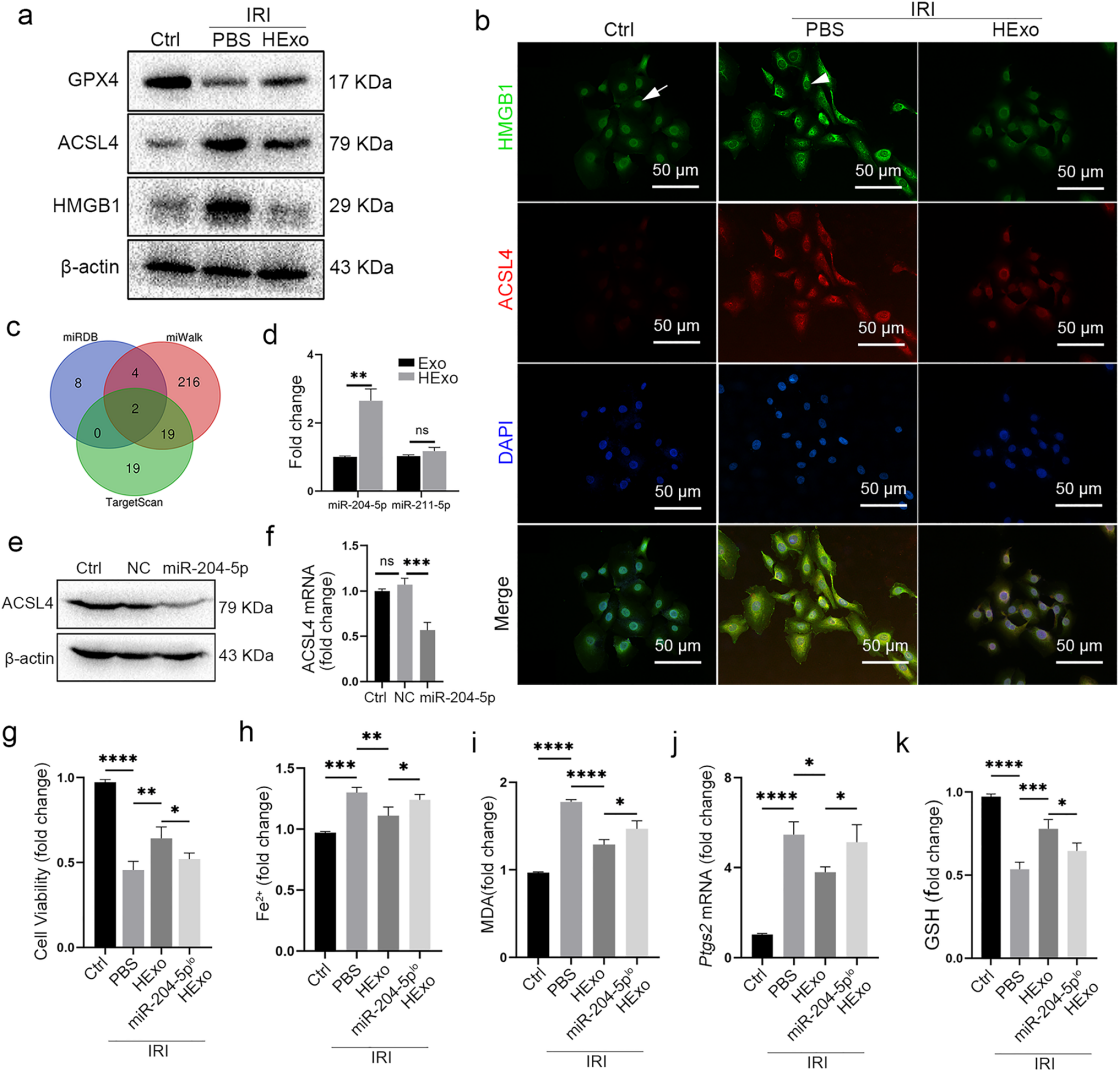
HExos inhibit ferroptosis by delivering miR-204-5p. An in vitro model of IRI was established, and HExos were administered. **a** Protein levels of GPX4, ACSL4, and HMGB1. **b** Expression level and subcellular localization of HMGB1 (green) and ACSL4 (red). HMGB1 was released from the nucleus (white arrow) to the cytoplasm (white arrowhead) when exposed to IRI. **c** Venn diagram showing the number of miRNAs targeting ASL4 predicted by TargetScan, miRWalk and miRDB databases. **d** Relative miR-204-5p and miR-211-5p levels in Exos and HExos. **e**, **f** Western blot (**e**) and qRT-PCR (**f**) verified the downregulating effect of miR-204-5p on ACSL4. **g**–**k** PBS, Exos, or miR-211-5p^lo^ Exos were administered in a cell model of IRI. The levels of cell viability (**g**), Fe^2+^ (**h**), MDA (**i**), *Ptgs2* mRNA (**j**), and GSH (**k**) were detected. Data are presented as the means ± standard deviation. The experiments were repeated three times. *P < 0.05, **P < 0.01, ***P < 0.001. *Exo* exosome, *miR-211-5p*^*lo*^* Exo* exosome with low level of miR-204-5p

To further understand the underlying mechanisms of HExos alleviation of ferroptosis, in vitro IRI model was established and HExos were administered. Consistent with in vivo studies, the HExos alleviated IRI-induced ferroptosis, which was manifested by the upregulation of GPX4 and downregulation of ACSL4 and HMGB1 at the protein level (Fig. 5a, b). Furthermore, HExos alleviated ferroptosis induced by erastin (Fig. S4).

Exosomal miRNAs are identified as a cell-to-cell communication mediator to perform biological functions. The miRNA databases miRDB, miWalk and TargetScan were used to predict miRNAs targeting ACSL4, and identified two miRNAs: miR-204-5p and miR-211-5p (Fig. 5c). Exos and HExos were isolated from MSC or HMSC culture medium, and qRT-PCR verified that miR-204-5p expression was significantly higher in the HExos than in the Exos (Fig. 5d). The miR-204-5p mimics effectively reduced ACSL4 expression at protein and mRNA level (Fig. 5e, f). Dual luciferase reporter assay showed that miR-204-5p inhibited luciferase activity in cells with the *ACSL4* WT plasmid compared with cells with the *ACSL4* MUT plasmid, indicating that *ACSL4* was a target of miR-204-5p (Fig. S5).

The HMSCs were transfected with miR-204-5p inhibitor to obtain exosomes with low miR-204-5p levels (miR-204-5p^lo^ HExos) (Fig. S6). miR-204-5p knockdown weakened the therapeutic efficacy of HExos in terms of cell viability and the levels of Fe^2+^, molecular markers of ferroptosis (MDA and *Ptgs*2 mRNA), and GSH in the IRI cell model (Fig. 5f–j). In addition, the anti-ferroptotic efficacy of the HExos was significantly weakened when Triton X-100 and RNase were utilized to dissolve the lipid membrane and degrade exosomal RNA (Fig. S6).

### HExos protect steatotic grafts from biliary fibrosis

**Fig. 6:**
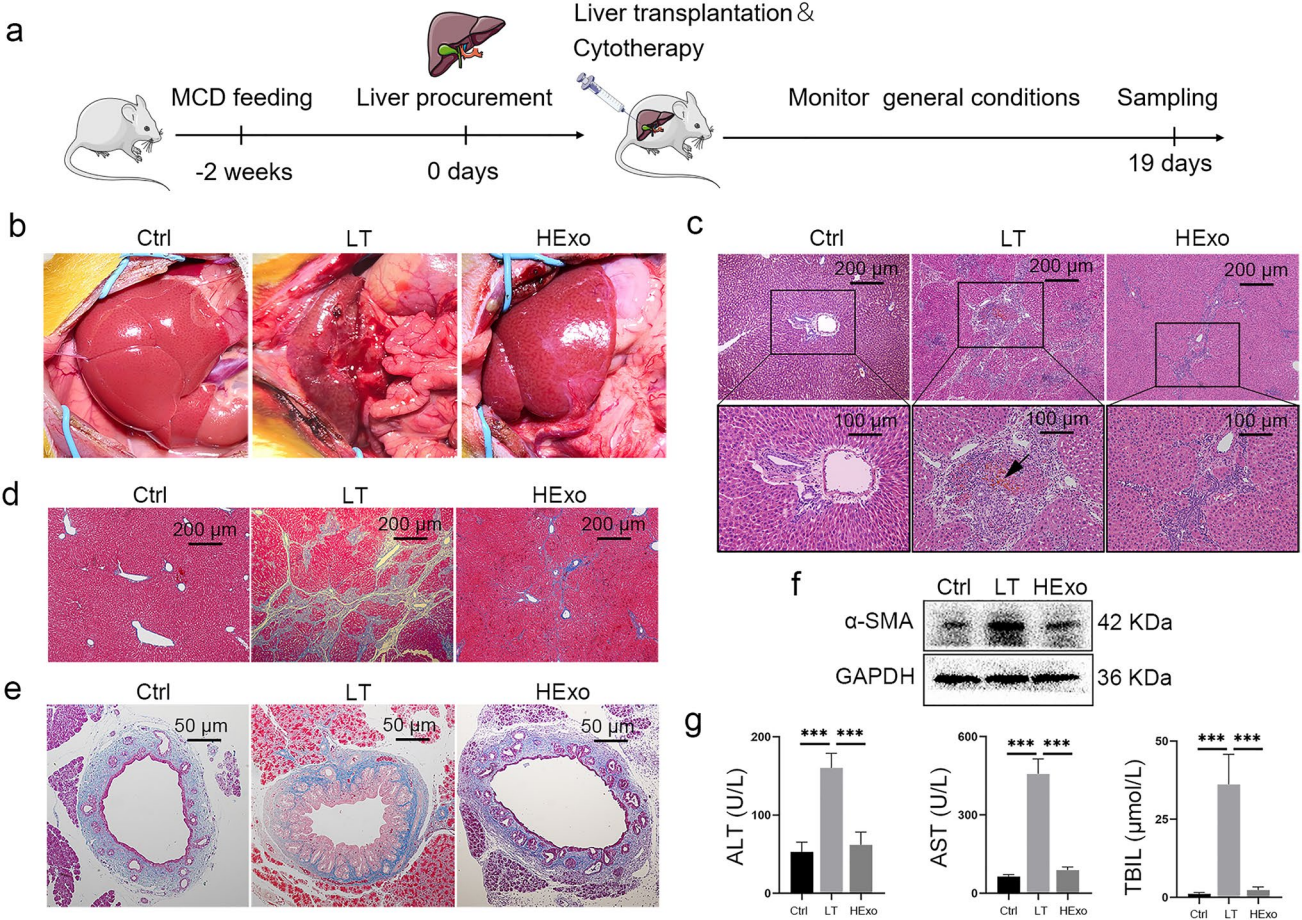
HExos alleviate biliary fibrosis of steatotic grafts. **a** Schematic diagram showing the observation of long-term biliary complications in the fatty LT rat model. **b** Gross appearance of livers 1 month after LT. **c** Representative HE staining images of livers. **d**, **e** Representative Masson staining images of liver (**d**) and common bile duct (**e**). f α-SMA level was detected by Western blot. **g** Liver functions (ALT, AST, and TBIL) were detected 1 month after LT (*n* = 6 mice per group). Data are presented as the means ± standard deviation. ***P < 0.001. *ALT* alanine aminotransaminase, *AST* aspartate transaminase, *TBIL* total bilirubin; HExo, exosome derived from HO-1-modified mesenchymal stem cells, *LT* liver transplantation

At the end of this study, we assessed long-term biliary fibrosis one month after LT (Fig. 6a). The recipients administered with PBS developed severe abdominal adhesion occurred, and the livers appeared dark red and swollen (Fig. 6b). Histology examination revealed biliary casting and bile embolism (Fig. 6c). Masson staining revealed intrahepatic and extrahepatic biliary fibrosis (Fig. 6d, e), confirmed by the high level of α-smooth muscle actin (α-SMA) (Fig. 6f), which is secreted by activated hepatic stellate cells. Additionally, liver function deterioration, especially increased TBIL level, was observed, suggesting the presence of cholestasis (Fig. 6g). The HExos improved hepatic histology and liver function, and alleviated biliary fibrosis (Fig. 6b–g).

## Discussion

Organ shortage and high mortality on waiting lists have led to the increased usage of ECD livers [22]. Moderate to severe macrosteatotic liver is a common type of ECD liver, which is often discarded because of its suboptimal quality. A meta-analysis of 10 studies demonstrated that moderate fatty liver (> 30%) increased graft dysfunction incidence by three times, while severe fatty liver (> %) increased the incidence by six times [23]. Hepatocyte swelling caused by lipid droplet accumulation leads to hepatic sinusoid narrowing and decreased capillary blood flow, and finally promotes the formation of a chronic hypoxic environment in fatty liver [23]. Compared with hepatocytes, biliary epithelial cells contain a lower level of antioxidants and are more vulnerable to IRI [24]. Consequently, steatotic grafts are at high risk of biliary complications, which remain one of the most significant factors affecting long-term outcomes of LT [24]. Although various methods to improve IRI have been developed, the treatment of biliary complications after LT remains challenging.

MSCs promote the repair of injured tissue by secreting exosomes [25]. In a previous study, we determined that HO-1 modification altered mRNA and miRNA profiles and increased the anti-inflammatory capacity of MSCs [26]. In the present study, we further investigated the role of HExos in improving biliary IRI of steatotic grafts, and identified a novel mechanism.

Recent studies suggested that ferroptosis is important in IRI and is a potential therapeutic target for IRI [27, 28]. Ferroptosis is a form of programmed cell death characterized by excessive iron-dependent lipid peroxidation [29]. During IRI, iron homeostasis is interrupted. Fe^2+^ accumulates and oxidizes lipids to generate large amounts of lipid-ROS through the Fenton reaction, which leads to cell damage. Meanwhile, GSH is depleted and the GPX4-dependent antioxidant system is inhibited. Essentially, ferroptosis is therefore a type of oxidative injury. ACSL4 promotes phospholipid biosynthesis and the accumulation of lipid peroxidation products, and is critical for ferroptosis. RNA-seq and analysis revealed that the ferroptosis pathway was enriched in the fatty LT model and that HExos administration rescued the upregulated ACSL4 expression. We speculated that miRNAs delivered by HExos bound to *ACSL4* mRNA to induce mRNA degradation and translational repression, and determined that miR-204-5p was abundant in HExos and reduced ACSL4 transcript and protein levels. The present results indicated that HExos attenuated ferroptosis to improve IRI in steatotic grafts by delivering miR-204-5p. However, numerous miRNAs have been detected in MSC-derived exosomes. A single miRNA can bind to hundreds of mRNAs and a single mRNA may be targeted by many miRNAs [30], suggesting that miR-204-5p may not be the only target-specific factor to regulate ferroptosis. Fan et al. [31] reported that miR-19a suppressed ferroptosis by targeting iron-responsive element-binding protein 2 (IREB2), another key pro-ferroptosis enzyme. Due to technical differences in exosome isolation or tissue source, definition of the categories and biological functions of exosomal miRNAs remain incomplete. Collectively, different miRNAs form complex networks to regulate biological processes and homeostasis [30].

During ferroptosis, the cell membrane integrity is eventually destroyed. Consequently, DAMPs released from the nucleus activate Kupffer cells, which are the major intrahepatic innate immune cells that regulate liver homeostasis and mediate liver injury. Activated Kupffer cells secrete large amounts of cytokines such as IL-1β and IL-6, establishing a pro-inflammatory microenvironment [32, 33]. IL-1β and IL-6 recruit T cells to the liver [32, 33]. This phenomenon partially accounts for how IRI exacerbates the transplantation rejection reaction. We determined that HExos reduced T cell recruitment in the liver by inhibiting Kupffer cell activation and pro-inflammatory cytokine release. Our study revealed a uniform mechanism of relevance between IRI and transplantation rejection. HExos protected against IRI in a fatty LT rat model and also alleviated the downstream immune injury, as confirmed by decreased CD4 + /CD8 + T cell ratio, a biological indicator of transplantation rejection [34].

**Fig. 7:**
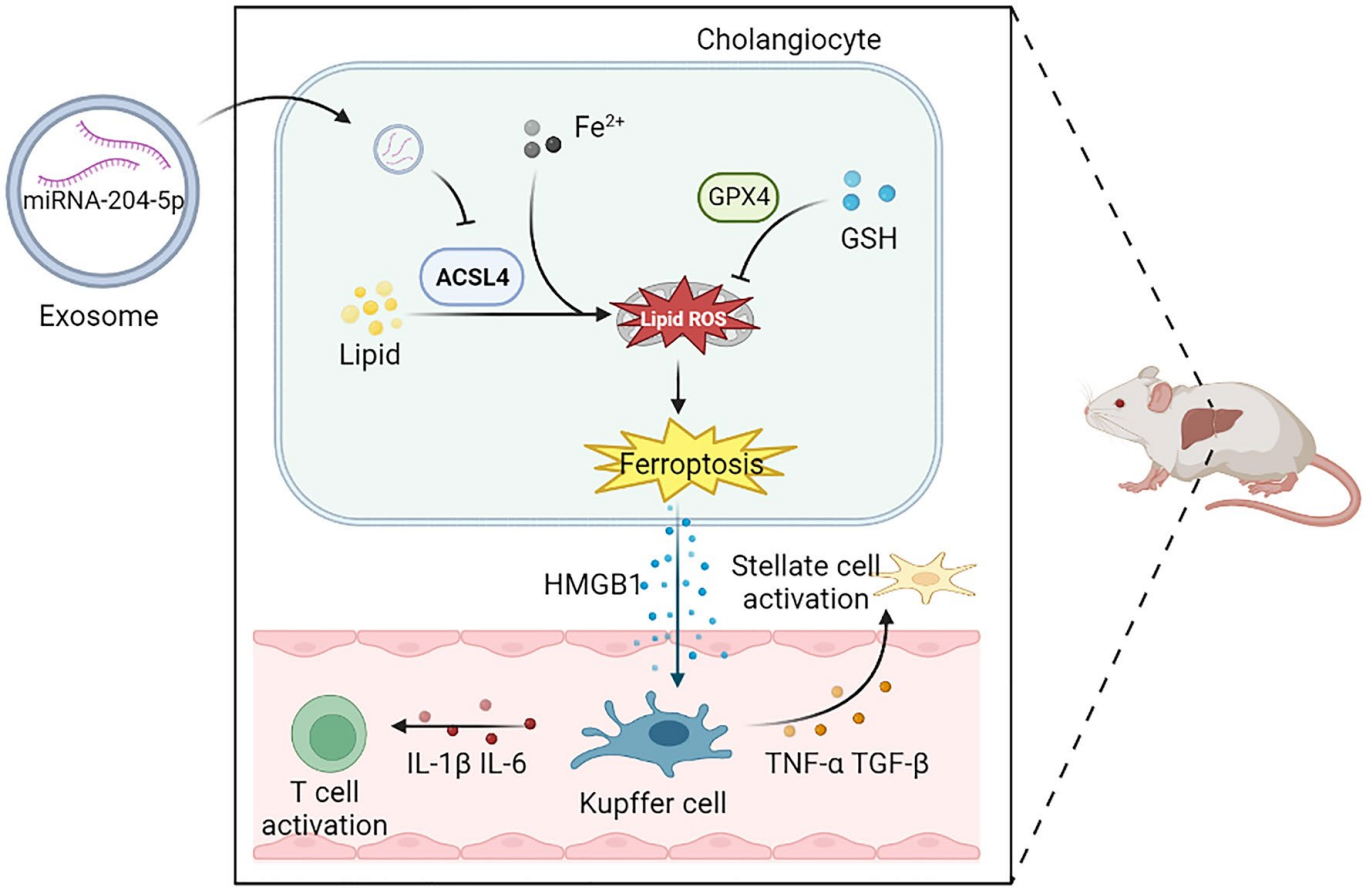
Schematic diagram of HExos attenuating biliary IRI. miR-204-5p derived from HExos inhibits ferroptosis through targeting ACSL4, thereby relieving inflammation induced by IRI and alleviating long-term biliary fibrosis after LT. *ACSL4* acyl-CoA synthetase long chain family member 4, *GPX4* glutathione peroxidase 4, *HMGB1* high mobility group box 1, *IL-1β* interleukin-1β, *IL-6* interleukin-6, *TNF-α* tumor necrosis factor-α, *TGF-β* transforming growth factor-β, *GSH* glutathione, *HSC* hepatic stellate cells. Figure 7 was constructed using pictures from Servier Medical Art (https://smart.servier.com)

On the other hand, Kupffer cells secrete pro-fibrotic cytokines such as TGF-β and TNF-α to activate hepatic stellate cells [35]. The deposition of extracellular matrix secreted by hepatic stellate cells around bile ducts is one of the main causes of biliary fibrosis, biliary stricture, and cholestasis after LT. Most animal studies on biliary injury assessed pathological changes 1–7 days after LT [36]. However, biliary complications occur within 12 months after LT in humans [37], which is comparable to 2 weeks in rats [38]. In this study, considering that biliary complications occur later, long-term biliary injury was assessed 4 weeks after LT. Our study is novel and different from other studies as we established a LT rat model that is closer to the real-world condition of biliary injury. Moreover, our study linked early IRI to long-term biliary fibrosis. HExos prevented initial IRI and DAMP release after LT by inhibiting ferroptosis, and relieved subsequential biliary fibrosis (Fig. 7).

Exosomes contain similar cargos as their parent cells and are easy to store and transport, which renders them an ideal cell-free therapy. We harvested exosomes with better therapeutic properties from HMSCs, and suggested a simple and economical method to prevent biliary injury in fatty LT and broaden the donor pool.

This study reported interesting findings. First, ferroptosis was implicated in IRI in steatotic LT. Next, Kupffer cells activated by ferroptosis linked IRI to downstream injury, such as transplantation rejection and biliary fibrosis. Finally, our main finding was that HExos prevented acute IRI and long-term biliary fibrosis by inhibiting ferroptosis. However, there are currently no validated and clinically relevant models of ischemic cholangiopathy after transplantation in rats. Therefore, the translation of these results to clinical setting requires further study.

## Conclusion

In summary, we found that ferroptosis was vital in IRI of fatty LT, and HExos effectively attenuated IRI and subsequent biliary fibrosis. Our findings provided new insights into the pathogenesis of IRI in fatty LT and identified a potential therapeutic strategy to safely utilize steatotic grafts.

### Supplementary Information

Below is the link to the electronic supplementary material.

Supplementary file1 (PDF 466 KB)

Supplementary file2 (PDF 187 KB)

## Data Availability

The raw RNA sequencing datasets reported in this paper have been deposited in Sequence Read Archive (PRJNA795289). The mRNA targets of relevant miRNAs were predicted by MiRDB (http://www.mirdb.org/), TargetScan (https://www.targetscan.org/), and miRWalk (http://mirwalk.umm.uni-heidelberg.de/). All other datasets used and analyzed during the current study are available from the corresponding author on reasonable request.
