# Supplementary material for: Exosomes derived from bone marrow mesenchymal stem cells alleviate biliary ischemia reperfusion injury in fatty liver transplantation by inhibiting ferroptosis
Source: Mol Cell Biochem. 2023 May 27;479(4):881–94. doi: 10.1007/s11010-023-04770-8 (PMC11016128; doi:10.1007/s11010-023-04770-8)
Supplement: Supplementary file 1 — Supplementary file1 (PDF 466 KB) [file 11010_2023_4770_MOESM1_ESM.pdf]

Exosomes derived from bone marrow mesenchymal stem cells alleviate biliary ischemia reperfusion injury in fatty liver transplantation by inhibiting ferroptosis

Xuan Tian[1], Longlong Wu[1], Xiang Li[2], Weiping Zheng[3,4], Huaiwen Zuo[2], Hongli Song[3, 5*]

Affiliation:

[1] School of Medicine, Nankai University, Tianjin, P.R. China

[2] Tianjin First Central Hospital Clinic Institute, Tianjin Medical University, Tianjin 300070, P.R. China

[3] Department of Organ Transplantation, Tianjin First Central Hospital, School of Medicine, Nankai University, Tianjin 300192, P.R. China

[4] NHC Key Laboratory of Critical Care Medicine, Tianjin, 300192, P.R. China

[5] Tianjin Key Laboratory of Organ Transplantation, Tianjin P.R. China

Correspondence to: Hongli Song, MD, Ph.D., Professor of Medicine, Department of Organ Transplantation, Tianjin First Central Hospital and Tianjin Key Laboratory of Organ Transplantation, No. 24 Fukang Road, Nankai District, Tianjin 300192, P.R. China. Phone: +86-22-23626928; Fax: +86-22-23626622; Email: hlsong26@163.com; songhl@tmu.edu.cn.

Supplementary Figures

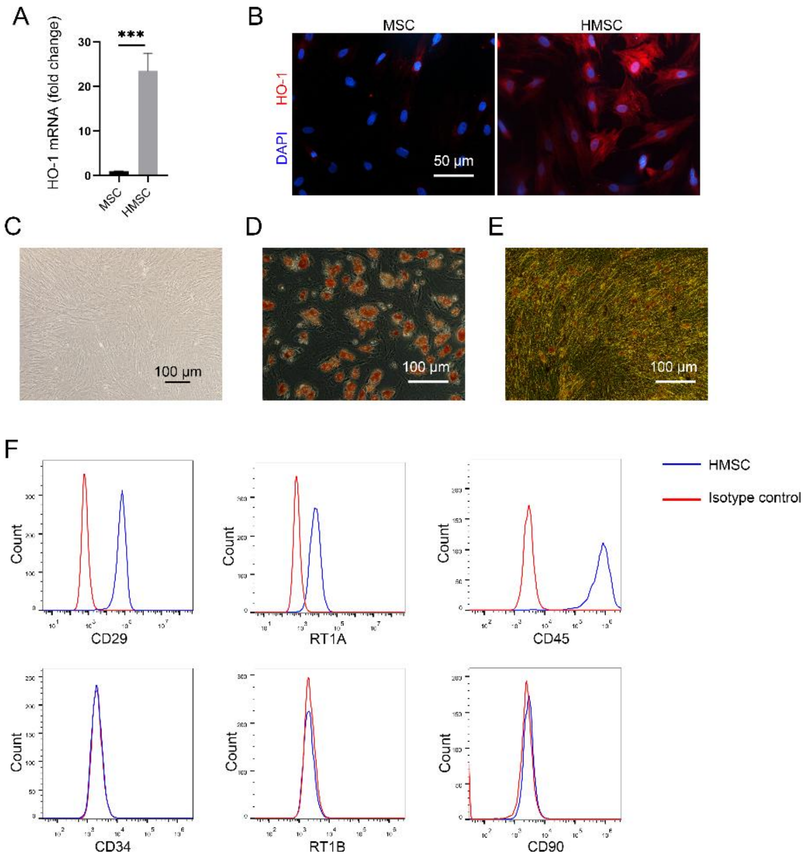

Figure S1 Preparation and identification of HMSCs.

A, B MSCs overexpressed with HO-1(HMSCs) were identified by qRT-PCR (A) and immunofluorescence (B). C-E HMSCs showed a long spindle shape arranged in swirls under light microscope (C), and could be induced to differentiate into adipocytes (D) and osteogenic cells (E). G Cell surface markers of HMSCs were detected by flow cytometry.

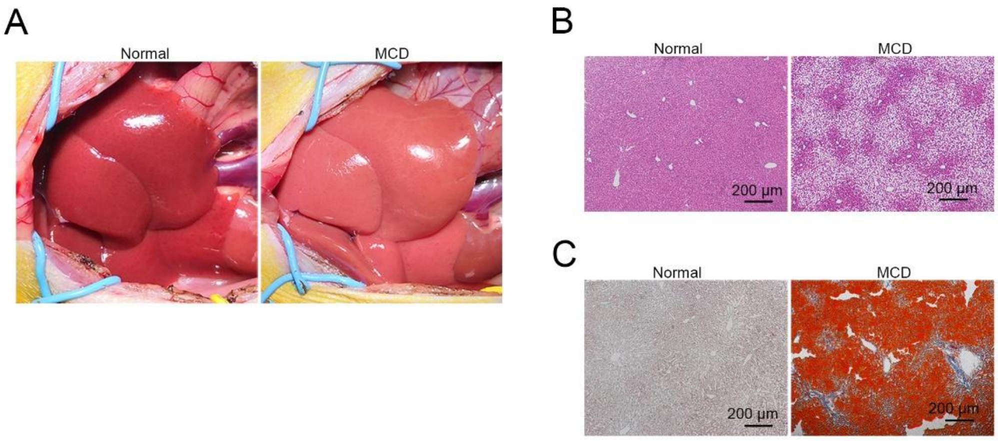

Figure S 2 Establishment of fatty liver rat model via MCD diet

A Gross appearance of fatty liver. Normal liver was characterized by reddish color with sharp edge. Steatotic liver was yellowish, greasy, fragile, and slightly enlarged, with round and blunt edge. B, C HE staining (B) and oil red O staining (C) showed accumulation of lipid droplets in MCD liver.

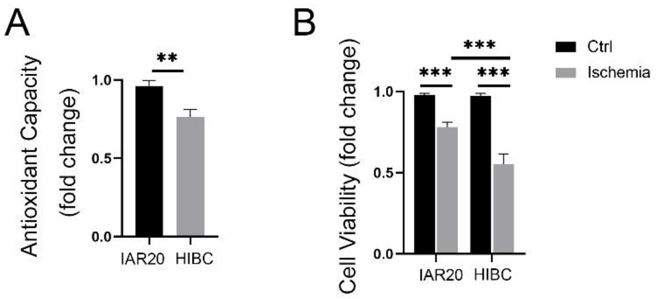

Figure S3 Cholangiocytes were more susceptible to IRI than hepatocytes.

A Antioxidant capacities of cholangiocytes (HIBCs) and hepatocytes (IAR20 cells) were detected using a total antioxidant capacity assay kit with a rapid ABTS method. B Hepatic IAR20 cells and HIBCs were exposed to hypoxia for 1 h (Ischemia group), or cultured in normal condition (Ctrl group), and cell viabilities were detected using a CCK-8 kit. HIBCs, Human intrahepatic bile duct epithelial cells.

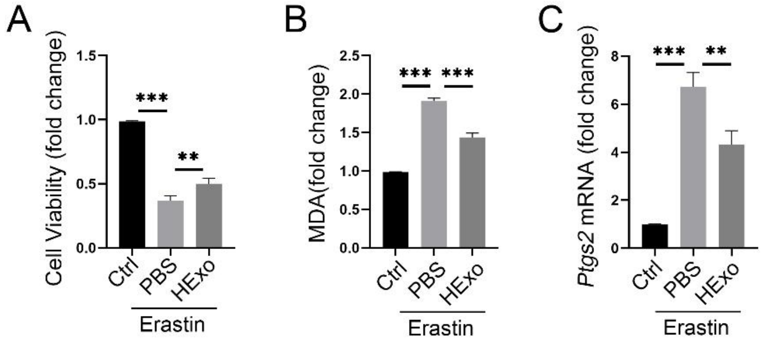

Figure S4 HExos alleviated ferroptosis induced by erastin.

HIBCs were treated with erastin (20 μM) to induce ferroptosis and cotreated with PBS or HExos (2.5×10^9 particles) for 6 h. Cell viability (A), malondialdehyde content (MDA; B), and *PTGS2* mRNA level (C) were determined.

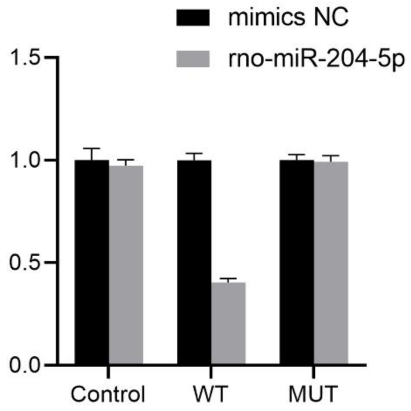

Figure S5 Dual luciferase reporter gene assay

Verification of targeted binding of miR-204-5p to the 3′-UTR of *ACSL4* mRNA in HEK 293T cells.

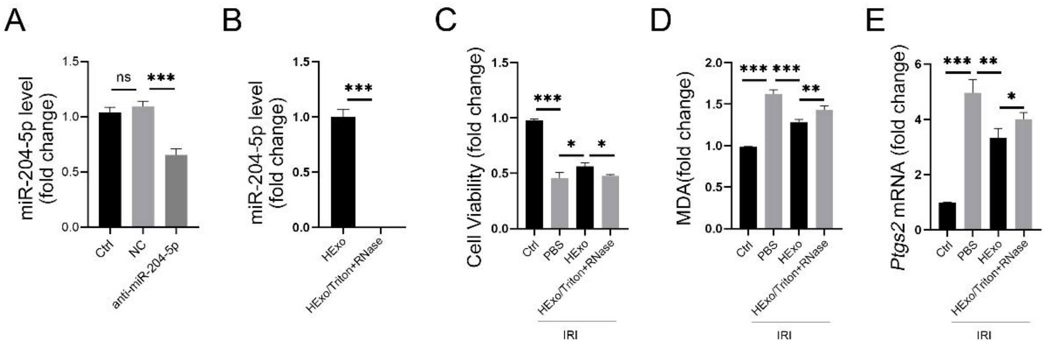

Figure S6 HExos inhibited ferroptosis by delivering miRNA.

A HMSCs were transfected with miR-204-5p inhibitor, and exosomes with low level of miR-204-5p (miR-204-5p^{lo} HExos) were extracted. miR-204-5p level of miR-204-5p^{lo} HExos was detected by qRT-PCR. B HExos were with Triton X-100 and RNase, and miR-204-5p level detected by qRT-PCR. C-E HIBCs were exposed to ischemia for 1 h and reperfusion for 2 h, and then co-cultured with PBS, HExos, or HExos /Triton X-100 + RNase for 6 h. Cell viability (C), MDA (D), and *PTGS2* mRNA (E) levels were detected.
