# Supplementary material for: Exosomes derived from bone marrow mesenchymal stem cells alleviate biliary ischemia reperfusion injury in fatty liver transplantation by inhibiting ferroptosis
Source: Mol Cell Biochem. 2023 May 27;479(4):881–94. doi: 10.1007/s11010-023-04770-8 (PMC11016128; doi:10.1007/s11010-023-04770-8)
Supplement: Supplementary file 2 — Supplementary file2 (PDF 187 KB) [file 11010_2023_4770_MOESM2_ESM.pdf]

Exosomes derived from bone marrow mesenchymal stem cells alleviate biliary ischemia reperfusion injury in fatty liver transplantation by inhibiting ferroptosis

Xuan Tian[1], Longlong Wu[1], Xiang Li[2], Weiping Zheng[3,4], Huaiwen Zuo[2], Hongli Song[3, 5*]

Affiliation:

[1] School of Medicine, Nankai University, Tianjin, P.R. China

[2] Tianjin First Central Hospital Clinic Institute, Tianjin Medical University, Tianjin 300070, P.R. China

[3] Department of Organ Transplantation, Tianjin First Central Hospital, School of Medicine, Nankai University, Tianjin 300192, P.R. China

[4] NHC Key Laboratory of Critical Care Medicine, Tianjin, 300192, P.R. China

[5] Tianjin Key Laboratory of Organ Transplantation, Tianjin P.R. China

Correspondence to: Hongli Song, MD, Ph.D., Professor of Medicine, Department of Organ Transplantation, Tianjin First Central Hospital and Tianjin Key Laboratory of Organ Transplantation, No. 24 Fukang Road, Nankai District, Tianjin 300192, P.R. China. Phone: +86-22-23626928; Fax: +86-22-23626622; Email: hlsong26@163.com; songhl@tmu.edu.cn.

Supplementary Tables

Table S1. Sequences of the primers

Primer	Sequence
ACSL4 FORWARD	TTCCGCTTGTGACTTTATATGCTACCC
ACSL4 REVERSE	AATAATGCCGCCTTCAGTTTGCTTTC
Ptgs2 FORWARD	CATTTGATTGACAGCCCACCAACTTAC
Ptgs2 REVERSE	AGTCATCAGCCACAGGAGGAAGG
HO-1 FORWARD	AGGAGATAGAGCGAAACAAGCAGAAC
HO-1 REVERSE	GCTGTGTGGCTGGTGTGTAAGG
IL-1β FORWARD	AATCTCACAGCAGCATCTCGACAAG
IL-1β REVERSE	TCCACGGGCAAGACATAGGTAGC
IL-6 FORWARD	AGTTGCCTTCTTGGGACTGATGTTG
IL-6 REVERSE	GGTATCCTCTGTGAAGTCTCCTCTCC
TNF-α FORWARD	ATGGGCTCCCTCTCATCAGTTCC
TNF-α REVERSE	CCTCCGCTTGGTGGTTTGCTAC
TGF-β FORWARD	GACCGCAACAACGCAATCTATGAC
TGF-β REVERSE	CTGGCACTGCTTCCCGAATGTC
β-actin FORWARD	CGCGAGTACAACCTTCTTGC
β-actin REVERSE	ATACCCACCATCACACCCTG
miR-204-5p FORWARD	GTCGTATCCAGTGCAGGGTCCGAGGTGCA CTGGATACGACAGGCAT
miR-204-5p REVERSE	TGCGGTTCCCTTTGTCATCCTA
miR-211-5p FORWARD	GTCGTATCCAGTGCAGGGTCCGAGGTGCA CTGGATACGACAGGCAA
miR-211-5p REVERSE	TGCGGTTCCCTTTGTCATCCTT
U6 FORWARD	CGCTTCGGCAGCACAT
U6 REVERSE	ATTTGCGTGTCATCCTTGC

Table S2. Sequences of miR-204-5p

Name	Sequence
no-miR-204-5p mimics	UUCCCUUUGUCAUCCUAUGCCU<br>GCAUAGGAUGACAAAGGGAAUU
no-miR-204-5p inhibitor	AGGCAUAGGAUGACAAAGGGAA
Mimics negative control	UUC UCC GAA CGU GUC ACG UTT<br>ACG UGA CAC GUU CGG AGA ATT
Inhibitor negative control	CAGUACUUUUGUGUAGUACAA

Table S3. Differentially expressed genes between Ctrl, LT and HEXO groups.

Gene Symbol	Ctrl1 TPM	Ctrl 2 TPM	Ctrl 3 TPM	LT1 TPM	LT2 TPM	LT 3 TPM	HEXO1 TPM	HEXO2 TPM	HEXO3 TPM
'Akr1d1'	1210.33	1152.1	1237.6	285.49	171.86	239.83	802.34	801.6	650.43
'Acnat2'	176.36	147.02	170.12	60.91	36.51	45.56	170.88	174.26	136.54
'Fabp7'	172.94	166.31	163.05	26.07	26.43	25.95	92.78	92	94.58
'Cyp3a18'	762.81	767.5	759.01	37.67	25.21	38.44	141.93	116.49	106.18
'Cyp2a2'	357.21	345.97	378.8	50.04	21.7	28.49	167.95	155.91	142.43
'Cd74'	296.57	295.11	270.92	1606.81	1713.55	1804.05	118.2	114.9	116.57
'RT1-A2'	210.9	210.52	217.24	920.41	1391.76	1067.69	125.46	101.95	120.59
'Cxcl9'	54.66	58.64	61.91	7122.27	7134.13	5603.35	74.08	54.47	79.86
'Lyz2'	27.55	27.23	24.13	2267.64	1527.92	2348.15	596.47	275.41	312.29
'Ubd'	34.57	32.06	23.91	4675.93	3868.17	4471.59	308.15	321.72	363.8
'A2m'	21.05	15.1	21.29	206.97	143.45	136.28	676.16	601.67	473.62
'Cxcl10'	8.78	9.37	5.94	893.74	778.95	768.82	21.37	17.7	19.42
'Ifi47'	6	6.2	3.86	1081.82	881.98	1066.78	40.17	33.7	33.4
'Psmb9'	13.31	13.82	14.3	544.16	572.26	580.75	36.7	33.11	34.69
'Stat1'	20.98	22.28	21.36	572.42	509.07	593.35	29.42	26.25	24.16
'Irf1'	10.26	8.9	9.96	340.89	361.42	317.44	32.62	27.5	33.07
'Igtp'	8.99	7.98	8.45	505.3	410.97	452.5	20.22	18.32	19.57
'Plac8'	9.4	11.27	9.74	474.42	418.01	493.03	26.5	23.34	25.22
'RT1-Db1'	40.89	42.84	42.14	241.86	253.83	233.29	13.83	13.85	16.02
'RT1-CE4'	23.61	28.5	24.35	174.92	249.08	212.63	23.91	18.74	26.1
'RT1-N2'	32.33	30.52	32.7	220.31	178.56	226.85	18.68	20.09	20.39
'Psmb10'	14.91	16.01	15.05	317.88	328.88	367.96	53.98	47.5	43.53
'Il18bp'	16.3	17.14	15.29	377.88	347.74	380.55	34.39	33.67	31.53
'Crip1'	26.1	18.82	23.58	198.31	276.44	209.5	45.28	31.72	45.26
'Ly6e'	25.72	29.43	30.16	345.05	265.64	358.06	64.44	48.42	47.69
'RT1-S3'	22.74	25.24	24.03	321.57	339.12	333.63	48.47	41.82	42.61

'RT1-A1'	65.89	62.82	64.29	314.91	457.44	400.31	45.6	37.71	48.45
'RT1-Da'	71.45	67.18	61.17	354.44	326.8	314.99	30.56	30.9	29.48
'Psme2'	59.31	59.84	49.92	372.92	377.41	356.57	71.33	70.83	70.76
'Psmb8'	26.48	31.56	28.97	575.68	611.45	634.16	57.4	53.89	60.56
'Uba7'	1.75	1.29	1.35	42.48	44.27	42.16	2.75	2.55	2.6
'LOC102555634'	0.7	0.95	1.06	45.79	32.92	38.07	3.47	3.44	2.57
'Oasl2'	1.29	1.16	1.06	39.63	28	31.43	4.3	3.51	3.63
'Zbp1'	0.65	0.58	0.79	19.31	26.71	23.28	1.29	1.28	1.33
'Sell'	0.41	0.86	0.68	21.98	19.79	24.23	1.77	1.39	1.98
'MGC105649'	0.61	0.42	0.55	21.61	16.32	25.31	2.89	1.63	1.26
'Herc6'	0.9	0.94	0.65	33.34	17.97	24.27	1.98	1.56	1.04
'Tnfrsf14'	0.95	1.05	1.13	26.66	19.96	23.08	2.19	1.66	1.85
'Ms4a6b'	0.97	0.76	1.27	28.84	21.71	22.02	1.99	1.91	1.33
'MGC105567'	1.12	0.77	0.87	41.4	29.92	35.32	2.1	1.72	1.25
'Lck'	1.42	1.27	1.82	24.21	31.55	25.7	2.65	1.45	2.38
'Pfkfb3'	1.12	1.3	1.21	24.89	23.31	20.98	2.86	1.13	1.41
'Pstpip1'	0.96	1.21	0.95	20.82	34.16	27.35	7.5	2.78	3.85
'Epsti1'	1.5	1.7	1.07	20.47	37.73	21.43	4.42	4.39	4.04
'Snx20'	1.38	1.89	2.11	17.57	34.97	20.61	5.02	2.99	4.92
'Pik3cd'	1.72	1.94	2.04	17.26	19.76	17.9	4.19	2.94	3.11
'Lcp2'	1.49	1.62	1.84	17.78	20.57	20.12	5.02	3.57	3.05
'Cd180'	1.68	1.58	1.66	22.69	14.55	18.99	4.19	3.16	2.47
'Tifab'	1.8	1.77	1.49	21.15	20.12	21.21	3.42	2.99	2.33
'Ugt1a2'	1.36	1.93	1.63	26.42	16.87	22.88	3.87	3.32	4.21
'Ripk3'	0.98	1.48	1.53	22.37	22.44	21.04	3.13	3.29	3.73
'Scimp'	1	1.88	1.05	24.6	20.95	21	4.42	2.97	2.96
'Arid5a'	1.04	0.73	0.82	14.82	18.7	16.42	4.93	2.91	2.7
'Dram1'	0.9	0.79	0.85	20.7	19.47	17.1	4.87	3.39	3.21
'RT1-M3-1'	1.46	1.07	0.79	21.5	23.33	22.98	3.57	2.25	2.74
'Slamf8'	0.61	0.74	0.87	24.35	23.7	26.18	3.71	2.44	3.58
'LOC102546968'	2.9	1.88	2.63	22.86	41.01	30.59	2.22	1.33	3.31
'Pycard'	4.36	3.3	2.03	19.99	22.36	18.22	3.29	4.54	2.61
'Vcam1'	2.86	2.51	2.73	20.03	16.7	17.8	5.26	2.58	2.33
'Cd274'	2.49	2.04	2.68	27.46	21.85	20.21	3.02	2.88	2.82
'Inpp1'	2.35	3.17	3.09	20.29	16.2	15.05	2.77	3.03	3.06
'Samhd1'	2.39	2.13	2.46	17.67	15.5	16.71	3.49	3.21	2.57
'Fgl2'	2.27	2.07	2.21	18.9	21.64	16.5	2.89	2.43	2.2
'Slc16a3'	0.08	0.2	0.09	14.29	17.57	18.18	3.81	2.51	3.26
'Batf'	0.44	0.86	0.23	16.33	27.11	17.62	2.62	1.51	2.58
'Cd8b'	0.31	0.3	0.24	17.69	20.33	16.8	2.01	1.15	1.13
'Kcnn4'	0.63	0.23	0.41	13.05	19.69	13.63	2.58	1.16	2.06
'Slc2a6'	0.29	0.16	0.13	10.09	14.02	9.63	1.38	1.28	1.22
'Bst1'	0.2	0.13	0.36	12.7	11.4	13.22	2.25	1.81	2.16
'Bcat1'	0.07	0.03	0.04	10.78	9.87	11.89	2.17	1.43	1.54

'Cd40'	1.01	0.8	0.58	12.25	11.14	9.87	1.78	2.01	2.67
'Snx10'	0.87	0.47	0.49	13.73	10.77	11.01	2.2	1.83	2.64
'Kcnab2'	0.83	0.63	0.41	8.97	10.62	9.51	1.73	1.64	1.74
'Runx3'	0.64	0.86	0.67	8.61	11.99	9.96	1.75	1.58	1.75
'Lat2'	0.49	0.6	0.57	9.16	10.72	13.23	1.86	0.65	1.98
'Il21r'	0.4	0.8	0.69	10.88	11.43	12.27	1.99	1.19	1.55
'Hist1h2ao'	1.11	5.65	1.75	8.39	15.52	19.66	1.17	1.64	2.44
'Gimap5'	1.15	2.53	2.43	14.85	18.39	11.24	2.42	1.53	2.09
'Ifit3'	0.91	1.87	1.68	17.04	19.9	13.93	1.8	1.16	1.51
'Map4k1'	1.21	1.72	1.7	13.95	17.14	16.13	2.07	1.25	2.17
'Dennd1c'	2.08	2.21	2.23	13.08	12.37	14.24	3.1	1.59	1.82
'Tnfaip8'	1.65	1.9	1.46	12.87	13.12	14.58	3.14	1.74	2.41
'Cenpj'	1.89	1.93	1.97	13.98	12.15	13.14	3.06	2.23	2.23
'Gimap4'	1.76	2.42	1.91	17.66	20.42	21.06	1.89	1.74	2.37
'Septin1'	1.99	2.09	2.24	13.81	18.94	16.7	1.67	1.52	1.83
'Samd9l'	1.94	1.68	2.21	23.64	14.51	20.15	2.3	1.73	1.97
'Il15ra'	2.3	2.58	3.05	17.17	14.16	15.94	2.23	1.39	1.54
'Ifi44l'	2.28	2.19	1.92	20.35	14.97	15.85	2.15	1.81	1.3
'Myo1g'	1.04	1.56	0.93	15.33	17.29	15.47	2.83	1.48	1.83
'Ptpn7'	0.87	0.97	0.92	11.99	16.57	14.5	3.19	1.54	1.56
'Themis2'	1.39	1.28	1.05	13.71	14.56	12.52	2.76	3.07	2.66
'Ikzf1'	1.43	1.65	1.16	12.75	12.77	12.12	3.41	2.32	1.94
'Sash3'	2	1.21	1.19	11.94	15.71	13.85	2.48	1.71	1.59
'Sla'	1.38	0.79	1.8	14.85	13.42	12.87	3.21	1.99	1.83
'Akna'	1.64	1.48	1.62	12.87	16.63	14.06	3	1.94	2.35
'Arhgap30'	1.11	1.51	1.47	13.51	17.53	14.8	3.29	1.97	2.01
'Rhoh'	1.38	1.6	1.25	10.88	10.04	12.96	0.85	1.8	1.55
'Cxcl14'	2.11	1.96	1.19	7.73	14.18	10.36	1.72	1.58	1.66
'Tcf7'	1.37	1.57	1.83	8.61	9.22	9.63	2.2	1.68	1.5
'Amigo2'	2.08	1.99	2.25	11.19	11.06	10.75	2.12	1.62	2.27
'LOC100366216'	1.45	1.09	2	12.5	9.28	12.73	1.42	0.45	0.92
'Dennd2d'	1.57	1.47	1.18	12.18	11.03	11.61	1.8	1.07	1.22
'Ccr5'	1.19	1.38	1.16	16.04	11.03	13.28	2.18	0.9	0.76
'Serpinb6b'	1.21	1.32	1.13	9.11	8.78	8.69	0.56	1.06	0.67
'Cyp26a1'	0.69	0.77	0.83	8.03	6.8	8.53	0.66	0.68	0.91
'Tbc1d10c'	0.75	0.63	1.12	7.12	8.48	7.81	0.61	0.58	0.83
'Tmem150b'	0.66	0.48	0.69	7.89	6.53	7.84	1.43	1.09	1.3
'Trim30'	0.9	0.97	0.9	8.89	6.88	8	1.42	0.85	0.95
'Layn'	1.02	0.47	0.65	7.6	8.87	7.62	1.94	1.16	1.47
'RGD1307182'	0.79	0.53	0.87	7.98	7.41	7.91	2.61	1.06	1.09
'Eif4e3'	0.92	1.06	0.96	8.3	7.9	9.9	1.9	1.64	1.45
'Fbxw17'	0.48	0.81	0.77	8.9	7.26	8.41	2.01	1.6	0.99
'Ccdc88b'	0.83	0.8	1.15	7.28	12.29	8.69	1.29	1	1.2
'Septin6'	0.91	1.1	0.9	10.35	12.44	12.94	1.78	0.83	1.5

'Cmpk2'	0.91	1.17	0.96	11.43	10.22	10.04	1.61	1.01	0.86
'Cytip'	1.24	1.48	1.11	10.41	11.31	11.65	1.33	0.7	1.03
'Mx1'	0.87	1.35	0.96	11.9	12.9	10.43	1.26	0.73	0.81
'Gzmm'	1.26	2.47	1.44	14.41	21.57	14.37	1.19	1.14	0.63
'Samd9'	1.05	0.95	1.07	22.17	16.75	19.04	1.49	1.18	1.14
'Ciita'	1.31	0.95	1.02	23.64	23.51	22.6	0.82	0.77	0.85
'Ctsw'	1.18	1.15	1.32	17.45	20.52	18.59	0.9	0.7	1.14
'Sult2a2'	1.06	1.18	1.38	10.29	19.13	11.66	0.33	0.31	0.39
'Acap1'	1.05	0.93	1.06	9.45	11.4	9.6	0.4	0.42	0.45
'RGD1565356'	0.76	0.89	1.05	11.26	9.98	10.88	0.54	0.42	0.25
'Cd3d'	0.19	0.75	0.4	16.48	18.36	17.26	0.4	0.57	0.52
'Cd27'	0.5	0.72	0.49	15.24	14.28	16.58	0.82	0.26	0.59
'Zap70'	0.44	0.47	0.58	10.12	14.12	12.51	0.53	0.39	0.69
'Fbxo27'	0.37	0.3	0.57	12.5	12.02	10.77	0.89	0.54	0.89
'Cst7'	0.55	0.74	0.62	14.4	18.39	16.21	1.08	1.09	1.04
'Pfkp'	0.54	0.84	0.73	11.36	12.77	11.59	1.12	1.13	0.93
'Dusp2'	0.85	0.66	0.59	12.28	11.53	11.35	1.29	1.06	1.44
'Nlrc5'	0.51	0.58	0.55	12.18	11.31	12.45	1.26	1	1.16
'Slpi'	0	0.19	0.21	54.39	46.9	76.56	18.59	3.72	5.17
'Acod1'	0.03	0.03	0	59.56	55.3	58.46	3.3	2.45	3.17
'Fcnb'	0.54	0.61	0.65	82.47	81.36	92.19	7.15	3.53	7.07
'Pla2g2a'	1.08	0.9	0.48	30.65	60.8	52.57	5.28	2.88	7.08
'Xcl1'	0.85	0	2.66	81.08	103.34	84.46	0	0	0.73
'Gbp6'	0.52	0.32	0.41	126.1	105.21	113.81	1.42	1.16	1.24
'RGD1305184'	0.21	0.09	0.15	106.84	76.58	101.23	0.59	0.56	0.5
'LOC102554096'	0.38	0.23	0.25	88.73	71.78	74.39	1.02	1.09	0.74
'Rpl30'	0	0.77	0	93.83	85.37	74.73	0.78	0	0
'Batf2'	0.74	1.25	0.57	71.58	75.9	62.71	1.2	0.87	0.75
'Socs1'	0.42	0.41	0.35	59.75	58.86	58.75	1.06	0.5	1.16
'Gzmbl2'	0.19	0.37	0	47.37	50.64	50.56	0.29	0.37	0.17
'Gzmb'	0	0	0	34.79	34.24	32.78	0	0	0
'Nos2'	0.07	0.07	0	19.82	35.22	15.03	0.22	0.27	0.53
'LOC100910934'	0.31	0.3	0.16	25.3	15.01	23.98	0.76	0.13	0.13
'LOC497963'	0	0	0	28.31	16.61	27.61	0.56	0.23	0.21
'Nkg7'	2.17	2.26	2.42	57.61	52.27	63.9	2.25	1.14	0.78
'Gzma'	1.48	1.15	2.01	74.06	91.47	69.96	0.92	0.44	0.67
'Lag3'	2.06	1.41	1.13	30.26	39.18	39.52	0.69	0.6	0.82
'Klrk1'	1.72	2.19	1.74	32.25	28.77	28.41	1.7	0.94	1.33
'Il2rb'	1.5	1.63	1.56	22.99	25.91	30.53	1.31	0.69	0.91
'Apol11a'	0.63	0.68	0.55	30.36	27.85	26.48	1.45	1.12	0.65
'Gbp4'	0.31	0.45	0.49	39.74	28.35	34.01	0.73	0.46	0.75
'Cd2'	0.97	1.38	1.3	23.51	35.1	28.37	0.74	0.96	0.97
'Gimap7'	1.3	1.35	1.04	23.1	22.09	22.92	0.56	1.44	0.49
'Cd3g'	1.03	1.86	1.35	27.91	25.24	23.99	0.91	0.22	0.78

'Cd3e'	0.75	0.92	0.73	24.99	22.29	26.96	0.41	0.4	0.32
'Prf1'	0.97	0.68	0.82	21.87	27.85	24.53	0.57	0.36	0.46
'Gsta1'	0	0	0	0	0	65.81	0	0	0
'Sh2d2a'	0.42	0.12	0.44	11.54	17.38	12.54	0.19	0.18	0.27
'Ifng'	0	0	0	15.66	12.5	11.21	0	0	0
'Defb52'	0	0	0	9.49	10.91	5.85	0	0	0
'Cd5'	0	0.31	0.34	7.04	9.71	8.27	0	0.04	0
'Tigit'	0	0.08	0.18	6.92	7.75	7.24	0	0.08	0.08
'LOC100911693'	0	0	0	7.96	12.94	11.32	0.7	0	0
'Zbtb32'	0.1	0.03	0.18	7.3	10.33	8.71	0.37	0.12	0.16
'Calhm6'	0.19	0.23	0.41	8.32	9.44	10.49	0.4	0.06	0.26
'Ms4a4c'	0.24	0.23	0.41	9.39	11.52	8.97	0.22	0.09	0.08
'Saal1'	0.13	0.04	0	8.45	8.33	7.22	0.93	0.71	0.61
'Tnip3'	0.13	0.13	0.28	6.65	8.32	5.63	0.61	0.73	0.61
'Glipr2'	0.19	0.27	0.49	7.26	8.44	7.63	1.65	0.28	0.93
'Card11'	0.41	0.28	0.2	7.19	10.79	9.71	0.86	0.38	0.64
'Slfn1'	0.38	0.46	0.49	9.91	7.58	10.82	0.93	0.42	0.66
'Hk2'	0.29	0.3	0.2	9.51	8.81	8.87	1.4	0.82	0.81
'Trem3'	0.28	0	0.51	6.54	4.3	7.36	0.49	0.12	0.52
'Cd247'	0.27	0.5	0.45	8.92	9.25	9.6	0.33	0.31	0.39
'Tbx21'	0.54	0.38	0.52	6.67	8.35	8.32	0.44	0.32	0.49
'Cxcr3'	0.66	0.81	0.52	7.14	6.65	9.13	0.34	0.49	0.5
'Icos'	0.45	0.65	0.8	7.55	6.49	7.51	0.57	0.32	0.2
'Gzmk'	1	1.22	0.26	4.22	6.59	4.25	0.65	0.37	0
'Sirpd'	0.62	0.95	0.84	5.65	4.24	4.94	0.18	0	0.24
'RT1-DOb'	0.6	1.6	0.88	7.58	4.8	5.39	0.16	0.35	0.31
'LOC100912658'	0.24	0.32	0.25	7.49	4.14	5.61	1.77	0.96	0.44
'Arl5c'	0.29	0.52	0.13	5.68	5.17	5.76	1.49	0.43	1.22
'Lpcat2'	0.42	0.52	0.36	5.39	6.02	4.89	1.19	0.74	0.72
'Zc3h12d'	0.36	0.33	0.14	5.05	6.22	5.02	1.01	0.76	0.72
'Cd244'	0.74	0.35	0.65	8.2	6.84	7.02	1.24	0.87	0.47
'Tagap'	0.36	0.55	0.37	7.74	6.91	7.14	0.74	0.59	0.51
'Sema4d'	0.51	0.56	0.41	8	7.65	8.26	0.91	0.91	0.73
'Cd200'	0.85	0.87	0.77	6.43	6.11	6.8	1.23	0.42	0.8
'LOC103690031'	0.8	1.06	0.74	6.94	5.04	6.62	1.27	0.48	0.8
'Aoc1'	0.86	0.58	0.76	6.12	5.32	7.14	0.89	0.55	0.66
'Itgb7'	0.78	0.47	0.47	6.59	7.24	7.47	0.94	0.57	0.71
'LOC308990'	0.52	0.54	0.68	4.89	6.03	6.91	1	0.99	0.75
'Plcg2'	0.52	0.52	0.72	5.29	5.36	5.22	1.05	0.6	0.89
'Rgcc'	0.37	0.36	0.64	6.37	9.2	5.13	0.51	0.85	1.56
'Unc13d'	0.65	0.92	0.69	3.9	5.71	4.55	1.4	0.73	0.71
'Prkcb'	0.96	0.7	0.63	5.8	7.29	6.11	1.29	1.16	1.08
'Traf3ip3'	0.5	0.67	0.51	5.27	9.74	6.68	1.18	0.86	0.9
'Ccrl2'	0.74	0.68	0.28	4.6	3.53	3.91	0.5	0.86	0.5

'Tlr4'	0.35	0.44	0.5	4.57	3.42	3.63	1.04	0.6	0.37
'LOC103692066'	0.8	0.72	0.68	5.41	3.41	3.5	1.07	0.67	0.65
'Gpr65'	0.41	0.3	0.21	3.23	2.9	2.92	0.32	0.4	1.13
'Il10'	0.06	0.42	0.26	2.92	3.41	2.58	0.61	0.97	0.76
'S1pr4'	0.31	0.41	0.2	2.82	3.4	2.7	0.52	0.23	0.28
'Fbxo39'	0.47	0.4	0.18	3	3.44	2.59	0.3	0.17	0.42
'Carmil2'	0.5	0.44	0.43	2.73	3.55	3.61	0.41	0.25	0.27
'Satb1'	0.26	0.54	0.49	2.92	2.86	3.18	0.36	0.1	0.39
'Scube3'	0.17	0.22	0.28	2.64	3.49	3.28	0.52	0.34	0.6
'Irf4'	0.25	0.12	0.2	3.23	3.16	2.91	0.47	0.29	0.37
'Treml2'	0.2	0.2	0.61	2.94	3.19	2.85	0.74	0.45	0.32
'Smpdl3b'	0.43	0.19	0.49	2.45	2.77	2.9	0.4	0.38	0.35
'Rasgrp1'	0.28	0.47	0.46	3.97	3.63	3.13	0.4	0.52	0.42
'LOC102546864'	0.39	0.38	0.59	3.29	3.74	3.19	0.58	0.34	0.39
'Ripor2'	0.47	0.42	0.45	4.16	3.72	4.14	0.69	0.38	0.46
'Atp2a3'	0.48	0.72	0.67	4.02	3.29	3.87	0.53	0.36	0.44
'Itga4'	0.59	0.45	0.46	3.39	2.63	2.45	0.59	0.37	0.52
'Jaml'	0.57	0.63	0.33	3.01	2.07	3.29	0.73	0.5	0.4
'Stx11'	0.29	0.36	0.14	3.59	5.11	3.7	0.76	0.54	0.46
'Sema7a'	0.16	0.36	0.39	3.37	4.43	4.37	0.47	0.29	0.36
'Mcpt8l2'	0.13	0.65	0.28	3.44	4.73	3.81	0.83	0.26	0.12
'Pdpn'	0.1	0.16	0.15	3.01	2.54	4.26	1.39	0.43	0.4
'Tmem154'	0.46	0.28	0.35	3.97	3.01	4.09	0.67	0.66	0.54
'Pou2af1'	0.17	0.27	0.21	3.53	3.03	3.64	0.95	0.64	0.47
'Ccdc69'	0.33	0.44	0.14	3.9	2.58	3.25	0.75	0.48	0.54
'Ms4a4a'	0.12	0.26	0.22	3.85	2.57	3.46	0.65	0.16	0.29
'Gbp3'	0.15	0.17	0.13	4.7	2.64	4.32	0.46	0.27	0.7
'LOC100912485'	0	0.73	0.77	4.25	2.21	3.09	0	0	0.92
'Cd79al'	0.65	0.05	0.94	4.65	3.72	3.2	0	0.79	0.1
'Gvin1'	0.32	0.52	0.4	3.95	2.97	3.85	0.19	0.13	0.09
'Ccr7'	0.32	0.48	0.34	3.15	2.81	3.23	0.08	0.48	0.07
'Mcpt2'	0.16	0.16	0.34	3.52	3.5	3.79	0.17	0.48	0.15
'Adgrg5'	0.31	0.27	0.41	4.75	3.29	3.26	0.22	0.28	0.35
'Batf3'	0.21	0.37	0.95	4.98	6.46	4.19	0.13	0.29	0.08
'LOC690862'	0	0.12	0.91	5.81	6.33	5.64	0.26	0.25	0
'Skap1'	0.31	0.54	0.26	5.43	6.21	5.59	0.44	0	0.06
'Cd6'	0.37	0.42	0.38	6.36	7.82	6.73	0.15	0.21	0.11
'Ptpn22'	0.45	0.29	0.36	6.47	5.47	7.17	0.19	0.15	0.14
'Rab19'	0.14	0	0.07	7.78	4.63	6.22	0.07	0.35	0
'LOC691695'	0	0	0	5.38	5.79	5.62	0.22	0	0
'Gbp1'	0.03	0	0.05	6.79	5.37	6.44	0.05	0.15	0.12
'Ctla4'	0	0.05	0.05	6.16	5.06	6.25	0.05	0	0
'Gzmbl3'	0	0	0	3.68	6.05	4.99	0	0	0
'Il2ra'	0.12	0.11	0.25	4.9	5.68	5.07	0.24	0.12	0.11

'Cd69'	0.06	0.17	0.36	5.73	4.63	4.27	0.18	0	0.16
'Pdcd1'	0.09	0	0.06	4.3	4.57	4.08	0.02	0.05	0.05
'LOC687532'	0.05	0.1	0.05	4.36	4.09	4.45	0.16	0.2	0.28
'Gpr171'	0.12	0.21	0.14	4.07	4.93	4.65	0.22	0.12	0.19
'Ocstamp'	0.4	0.22	0.36	5.26	4.94	4.09	0.5	0.5	0.63
'Itprip'	0.41	0.34	0.63	3.9	4.82	3.35	0.64	0.52	0.56
'Def6'	0.19	0.37	0.4	4.2	7.53	5.14	0.39	0.37	0.69
'Klrb1a'	0.51	0.5	0.75	3.69	5.44	5.28	0.32	0.1	0.37
'Fcho1'	0.67	0.49	0.56	4.48	6.82	6.28	0.6	0.3	0.56
'Rinl'	0.38	0.7	0.35	4.88	5.35	5.2	0.48	0.25	0.52
'H1f4'	0.39	0.57	0	2.32	6.71	3.85	0	0.58	0.88
'A1bg'	0	0	0	3.45	7.27	4.84	0.51	0.8	0.89
'Pacsin1'	0.32	0	0.06	3.67	5.44	3.51	0.71	0.63	1.1
'Loxl1'	0.57	0.31	0.37	2.28	2.39	2.69	13.65	6.74	9.54
'Trem2'	0	0	0.21	1.99	0.36	0.97	11.28	4.44	6.83
'LOC103690878'	0	0	0.13	1.1	0.56	0.7	5.47	5.32	4.77
'Gucy2c'	0.02	0.04	0	1.55	1.19	1.68	5.38	4.98	5.53
'Fndc1'	0.06	0.05	0.13	0.53	0.52	0.69	5.11	1.98	2.17
'Cxcl6'	0	0.14	0	1.3	0.82	0.24	4.2	1.29	5.83
'Adm2'	3.75	2.67	3.28	0.4	0.46	0.96	5.26	4.7	6.07
'LOC108348209'	4.17	4.11	2.5	2.22	0.33	0.68	3.25	2.89	4.11
'Slc39a5'	5.18	3.81	3.9	0.69	0.56	0.45	2.86	3.75	3.77
'Nat8f3'	3.39	3.37	3.73	0.2	0.14	0.02	0.54	0.51	0.32
'Abcg8'	3.94	3.85	3.71	0.32	0.28	0.27	1.47	1.73	1.69
'LOC501038'	3.68	4.01	3.41	0.57	0.55	0.68	2.24	2.77	1.8
'Erich5'	3.9	3.63	3.79	0.49	0.52	0.36	2.97	1.77	1.95
'Ackr4'	1.72	2.58	2.28	0.43	0.89	0.38	2.87	2.98	2.25
'Gfra1'	2.55	1.76	2.22	0.14	0.26	0.37	3.35	2.26	2.66
'Elmod2'	1.54	1.42	2.85	0.45	1.19	1.44	1.91	1.37	1.47
'Fmo2'	2.29	1.74	2.03	0.76	0.16	0.38	2.04	1.03	0.97
'Clec2dl1'	2.47	2.37	2.75	0.5	0.1	0.35	2.29	2.39	1.34
'Pmaip1'	0.2	0.24	0.21	0.74	4.75	3.01	0.21	0.1	0.09
'Prph'	0.67	0.1	0.35	0.61	4.22	1.32	0.11	0	0.24
'LOC100911215'	0.24	0.07	0.12	0.43	1.89	2.34	0.05	0	0.27
'LOC100909474'	0	0	0	0.51	1.43	1.64	0	0	0.38
'Gzmf'	0	0	0.13	0.29	1.38	0.78	0	0	0
'Lrrc23'	0	0	0.07	0.36	1.29	1.09	0.14	0	0
'Btnl2'	0.02	0	0	0.96	0.99	0.92	0.12	0.02	0.22
'Tox'	0.05	0.06	0.06	1.1	1.18	1.06	0.06	0	0.03
'Il12rb2'	0.11	0.08	0.03	0.85	1.11	1	0.07	0.07	0.03
'Fam169b'	0.1	0.05	0.05	0.74	1.17	0.98	0	0.1	0.09
'Nkain4'	0.16	0	0.22	0.64	1.16	0.91	0	0	0
'Ly9'	0.17	0.18	0.24	0.99	1.14	0.99	0	0.04	0
'Podnl1'	0.29	0.08	0.13	0.87	1.14	0.98	0.13	0.04	0.08

'Gata3'	0.1	0.1	0.3	0.82	1.88	1.31	0.09	0.14	0.2
'LOC102552360'	0.27	0.26	0.28	1.06	1.7	1.18	0.09	0.35	0.16
'Gjd3'	0.04	0.08	0.08	1.23	2.4	1.18	0.16	0	0.28
'Clec2d2'	0.05	0.17	0.04	1.07	1.76	1.14	0.16	0.04	0.48
'Zfp683'	0.15	0.2	0.05	1.02	1.59	0.73	0.21	0.1	0.14
'Lpo'	0.03	0	0	0.78	1.95	0.69	0.24	0.23	0.11
'Acp4'	0.14	0.33	0.29	1.72	1.56	0.83	0.26	0.18	0.21
'Cpa3'	0.4	0.19	0.07	1.77	1.32	0.75	0.07	0.13	0.48
'Eomes'	0.12	0.1	0.23	1.29	1.18	0.91	0.1	0.08	0.11
'Scin'	0.13	0.08	0.17	0.95	1.35	0.79	0.17	0.06	0.18
'Lpar5'	0.08	0.11	0.25	1	1.27	0.86	0.2	0.19	0.1
'Mcoln2'	0.1	0.03	0.07	1.47	1.54	0.85	0.14	0.03	0
'Cdca7l'	0	0.1	0.04	1.37	1.41	1.12	0.17	0.17	0.12
'LOC100911190'	0.05	0.03	0.02	1.4	1.15	0.96	0.17	0.15	0.19
'Pvrig'	0.23	0.09	0.05	1.52	1.02	1.2	0.24	0.32	0.12
'Foxp3'	0.14	0.1	0.07	1.5	1.2	1.5	0.34	0.17	0.13
'Runx2'	0.22	0.15	0.08	1.32	1.09	1.62	0.18	0.11	0.13
'Cd226'	0.14	0.13	0.15	1.43	1.19	1.41	0.16	0.11	0.17
'Gfi1'	0.18	0.11	0.11	1.04	1.49	1.58	0.11	0.04	0.07
'Sytl3'	0.12	0.04	0.08	1.34	1.71	1.62	0.08	0.08	0
'Sla2'	0.09	0.1	0.09	1.46	1.53	1.65	0.07	0.05	0.04
'Cma1'	0	0	0.23	1.5	1.37	1.33	0	0.21	0.1
'Gpr174'	0.05	0.13	0.14	1.7	1.32	1.27	0.04	0.05	0.03
'Ipcef1'	0.06	0.09	0.09	1.61	1.33	1.37	0.09	0.08	0.12
'Ly75'	0.07	0.05	0.07	1.62	1.35	1.56	0.17	0.1	0.14
'Ms4a18'	0.04	0.04	0.04	1.42	1.14	1.36	0.08	0.04	0.07
'Arpp21'	0.04	0.03	0.09	1.48	1.32	1.58	0.15	0	0.02
'Oas3'	0.13	0.1	0.14	1.56	2.64	1.32	0.19	0.41	0.31
'Timd4'	0.16	0.37	0.17	1.86	2.62	1.37	0.08	0.16	0.39
'Sytl1'	0.22	0.17	0.18	1.65	2.57	1.7	0.14	0.17	0.09
'Catip'	0.15	0.2	0.05	1.6	1.93	1.67	0.05	0.15	0.14
'Bcl11b'	0.08	0.09	0.17	1.73	2.08	1.45	0.16	0.1	0.03
'St8sia1'	0.06	0.13	0	1.59	2.63	1.82	0.13	0.13	0.23
'Camk4'	0.1	0.15	0.01	1.78	2.91	1.36	0	0.05	0.09
'Cd28'	0.02	0.05	0.08	1.78	2.02	2.01	0.31	0.3	0.07
'Slco4a1'	0.03	0.06	0.09	1.73	1.6	2.12	0.31	0.17	0.23
'Ddx60'	0.1	0.17	0.17	1.97	1.5	1.87	0.23	0.1	0.16
'Stk26'	0.1	0.15	0	1.99	1.84	1.24	0.3	0.14	0.09
'Kctd19'	0	0	0.03	2.08	1.44	1.53	0.09	0.06	0.16
'LOC108351974'	0	0.19	0	1.8	1.55	1.9	0	0	0
'LOC286960'	0	0	0	1.7	1.67	2.36	0	0	0
'Dtx1'	0.09	0.06	0.05	1.14	2.57	1.77	0.02	0.07	0.08
'Jakmip1'	0.04	0.19	0	0.96	2.05	1.61	0.04	0.15	0.1
'Ido1'	0	0	0	2.4	4.25	3.59	0	0	0

'Trav12-3'	0	0	0	2.28	4.11	2.13	0	0	0
'LOC102555392'	0	0.07	0.07	3.94	2.98	3.67	0.14	0.07	0
'Itk'	0.06	0.1	0.16	3.49	3.16	3.53	0.18	0.08	0.09
'Crtam'	0	0	0.06	3.91	3.96	2.4	0.11	0	0.05
'Tnfsf14'	0	0	0.2	3.58	4.58	3.09	0.28	0	0
'Steap1'	0.25	0	0.17	2.71	1.96	3.36	0.34	0.49	0.37
'Krt14'	0	0	0	2.36	1.49	3.83	0.13	0.15	0.5
'Pdcd1lg2'	0	0.02	0.12	2.55	2.02	2.27	0.06	0.06	0
'Ccr6'	0.06	0.14	0.15	2.28	1.96	2.48	0.09	0.06	0.12
'Slamf1'	0.06	0	0.07	2.77	2.86	2.71	0.06	0.09	0.06
'Scube1'	0.02	0	0	2.41	2.39	2.58	0.09	0.02	0.04
'Gzmbl1'	0	0	0	2.54	2.18	2.81	0	0	0
'Acta1'	0	0	0.14	1.77	2.64	3.75	0.14	0.13	0.06
'Il12rb1'	0.04	0.14	0.08	2.19	3.02	2.48	0.06	0.23	0.11
'Prkcq'	0.15	0.15	0.1	2.35	2.52	2.89	0.05	0.12	0.05
'Cmahp'	0.22	0.24	0.08	2.49	2.64	3.18	0.17	0.2	0.12
'Havcr2'	0.25	0.16	0.19	1.81	2.74	2.28	0.04	0	0.15
'Txk'	0.1	0.26	0.4	2.25	2.25	2.26	0.17	0	0.17
'Klrb1c'	0.34	0.33	0.63	2.19	2.55	2.64	0	0.08	0.14
'Sit1'	0.25	0.32	0.52	2.94	2.17	2.49	0	0.08	0.37
'Cd19'	0.36	0.22	0.34	2.57	1.89	2.49	0.11	0.14	0.15
'Gpc1'	0.05	0.02	0.03	1.64	2.76	2.3	0.37	0.26	0.48
'Gp6'	0.07	0.09	0	3.22	2.48	2.58	0.37	0.31	0.33
'Ly6h'	0	0	0	2.61	3.14	2.49	0.37	0.18	0.21
'Ms4a6c'	0.08	0.15	0.04	2.43	2.83	2	0.51	0.04	0.45
'Mcub'	0	0.23	0.11	2.38	2.97	2.44	0.78	0.27	0.28
'Sh2d1a'	0.29	0.19	0.21	2.02	3.06	2.49	0.17	0	0.09
'Ikzf3'	0.38	0.31	0.24	2.14	3.04	2.59	0.17	0.06	0.13
'Tnfrsf18'	0.32	0.1	0.13	2.55	3.07	2.43	0.1	0.1	0.18
'Hsh2d'	0.36	0.24	0.17	2.65	3.14	2.92	0.25	0.04	0.11
'Stk39'	0.11	0.17	0.09	2.31	3.7	3.08	0.26	0.12	0.16
'Stat4'	0.14	0.19	0.21	2.86	2.88	3.41	0.24	0.11	0.1
'Faslg'	0.22	0.28	0.3	2.76	2.33	2.4	0.4	0.34	0.26
'Slc38a1'	0.11	0.17	0.16	2.23	2.3	2.41	0.4	0.17	0.34
'Mefv'	0.11	0.27	0.11	2.05	2.44	2.23	0.47	0.37	0.29
'Olfm1'	0.37	0.04	0.08	2.5	2.14	2.4	0.18	0.19	0.25
'Lef1'	0.14	0.16	0.03	1.82	2.19	2.85	0.37	0.12	0.15
'Aim2'	0.15	0.3	0.21	2.73	2.14	2.76	0.32	0.15	0.05
'Lax1'	0.13	0.23	0.25	2.28	2.38	2.52	0.2	0.22	0.04
'Pde6h'	0.77	0.6	0	1.73	1.92	1.7	0.4	0	1.02
'Ms4a1'	0.11	0.39	0.41	2.63	1.33	1.5	0.42	0.17	0.15
'Klrd1'	0	0.42	0.3	2.63	1.76	0.95	0.3	0.21	0.06
'LOC103690099'	0	0.42	0	3.15	1.79	2.08	0.32	0	0.59
'Atp1a3'	0	0.02	0	2.16	1.58	2.27	0.51	0.25	0.3

'Atp10a'	0.06	0.05	0.1	2.25	1.82	2.36	0.61	0.29	0.37
'Gpr160'	0.17	0.28	0.12	2.26	1.32	2.08	0.36	0.24	0.42
'Ddr2'	0.2	0.21	0.22	2.51	1.67	2.43	0.59	0.25	0.13
'LOC102550614'	0.32	0.19	0.27	1.46	1.17	2.07	0.13	0.06	0.06
'Upk1b'	0.41	0	0.21	1.43	0.84	2.43	0.21	0	0.09
'Erp27'	0.1	0.31	0.44	1.86	1.23	2.47	0.32	0.1	0.09
'RGD1562652'	0.12	0.06	0.06	2.17	1.4	1.96	0.18	0.18	0.05
'Tnfrsf13c'	0.21	0.2	0.11	2.37	1.42	2.17	0.16	0.2	0
'Cd96'	0.13	0.3	0.09	1.59	1.34	1.74	0.09	0.17	0.16
'Reep2'	0.19	0.19	0.1	2.08	1.06	1.94	0.1	0.38	0.26
'Opn3'	0.36	0.3	0.33	1.91	2.09	1.73	0.43	0.46	0.19
'Klri1'	0.45	0.18	0.19	2.01	1.83	1.73	0.19	0.09	0.17
'Gpr132'	0.22	0.32	0.23	2.34	1.98	1.86	0.19	0.29	0.23
'Bank1'	0.3	0.3	0.25	2.15	1.6	1.83	0.15	0.22	0.17
'Tex13b'	0.13	0.16	0.06	1.06	1.7	2.16	0.16	0.3	0.26
'LOC108349511'	0.34	0.37	0.18	1.49	1.29	2.03	0.37	0.16	0.23
'Dusp4'	0.17	0.08	0.35	1.33	1.22	1.74	0.35	0.25	0.23
'LOC691320'	0.42	0.42	0.12	0.15	0	0	0.25	0.21	1.15
'Abcd2'	0.32	0.29	0.29	0.06	0.05	0.08	0.58	0.73	0.59
'LOC108349760'	0.28	0.2	0.29	0.02	0.04	0	0.48	0.41	0.63
'LOC100910033'	0.42	0.45	0.4	0	0	0.25	0.49	0.6	0.17
'Nim1k'	0.37	0.44	0.47	0.04	0	0	0.25	0.28	0.22
'LOC100912019'	0.4	0.67	0.63	0	0.04	0.08	0.42	0.4	0.29
'Ntsr1'	0.46	0.81	0.49	0.08	0.25	0.1	0	0.02	0
'Chst4'	0.04	0	0.04	0.23	0.27	0.43	0	0	0
'B3gnt5'	0.06	0.04	0.04	0.31	0.2	0.45	0.04	0.02	0.05
'Bnc1'	0.11	0	0.02	0.3	0.15	0.24	0.02	0	0.03
'Umodl1'	0	0.02	0.02	0.16	0.16	0.3	0.02	0	0.02
'LOC103689974'	0	0	0.04	0.27	0.14	0.4	0	0	0.03
'Ptpn5'	0	0	0	0.31	0.19	0.33	0.03	0	0
'Rsph4a'	0	0	0	0.35	0.17	0.15	0	0	0
'LOC102556096'	0	0	0.03	0.47	0.2	0.21	0	0.03	0.08
'Adgrd1'	0.05	0	0.02	0.42	0.12	0.37	0.1	0.02	0.06
'Kcnk7'	0.06	0	0	0.42	0.21	0.4	0	0.1	0.03
'Hap1'	0.03	0.05	0.09	0.43	0.28	0.35	0.03	0.03	0.06
'Lrrc37a'	0	0	0	0.08	0.04	0.06	0.01	0	0
'Otogl'	0	0.01	0.01	0.06	0.07	0.15	0	0.01	0
'Megf10'	0	0	0	0.12	0.04	0.13	0.01	0	0.01
'Crb2'	0.02	0	0.04	0.12	0.08	0.23	0.02	0	0.01
'Igfn1'	0.01	0	0.02	0.18	0.09	0.15	0	0	0
'Cysltr2'	0.03	0.02	0.03	0.22	0.22	0.22	0	0.03	0
'Fam78b'	0.03	0.05	0.03	0.24	0.31	0.18	0.03	0.03	0
'Camk2b'	0	0.05	0	0.02	0.25	0.23	0.02	0	0
'Nefh'	0.02	0	0	0.09	0.45	0.23	0.02	0.04	0.02

'Vgf'	0	0	0	0.13	0.42	0.09	0	0	0
'Slc5a9'	0.07	0.19	0.18	0	0	0.02	0.16	0.1	0.14
'Slc15a2'	0.06	0.06	0.07	0	0	0	0.09	0.06	0.06
'Ankrd63'	0.12	0.07	0.04	0.02	0.01	0.01	0.16	0.06	0.08
'Fam227a'	0.3	0.22	0.18	0	0.02	0.02	0.24	0.2	0.2
'Sgip1'	0.23	0.13	0.13	0.01	0.03	0.06	0.21	0.16	0.11
'Sertad4'	0.29	0.23	0.25	0.03	0.05	0.04	0.55	0.29	0.22
'Hrk'	0.16	0.2	0.13	0.02	0	0.03	0.41	0.28	0.26
'Nos1'	0.07	0.12	0.14	0.02	0.02	0.04	0.41	0.24	0.41
'Ascl1'	0	0	0	0.37	0.63	0.77	0	0	0.11
'Prss39'	0	0	0	0.45	0.42	0.64	0.1	0.09	0.05
'Tspan32'	0.02	0.04	0.11	0.47	0.41	0.64	0.05	0.09	0.08
'Garem2'	0.06	0.06	0.1	0.48	0.53	0.64	0.03	0.06	0.06
'Misp'	0	0	0	0.23	0.66	0.43	0	0.07	0.06
'Cd160'	0	0	0	0.51	0.75	0.51	0	0	0
'Galnt3'	0.03	0.06	0.03	0.46	0.65	0.44	0.1	0	0.06
'Ccnb1ip1'	0.05	0	0	0.44	0.7	0.34	0.11	0	0.05
'Slc14a1'	0.13	0.05	0	0.39	0.36	0.29	0.07	0.02	0
'Ano9'	0	0	0.04	0.44	0.35	0.39	0	0.03	0
'Il31ra'	0.02	0.02	0	0.38	0.37	0.33	0.02	0.03	0.04
'Themis'	0	0.03	0	0.33	0.35	0.36	0	0	0
'Zfp296'	0.07	0.03	0.15	0.49	0.41	0.4	0.11	0.07	0.06
'Col22a1'	0.12	0.08	0.05	0.35	0.56	0.41	0.05	0	0
'Klrc3'	0.07	0.03	0.06	0.45	0.44	0.46	0.03	0	0.03
'Myb'	0.03	0.04	0.03	0.4	0.41	0.41	0	0	0
'LOC100911800'	0	0.05	0.05	0.37	0.44	0.45	0	0	0.05
'Has1'	0	0	0	0.46	0.5	0.4	0.09	0	0
'Rgsl1'	0.03	0.02	0.02	0.41	0.47	0.44	0.1	0.05	0.08
'Calml3'	0	0	0.07	0.76	0.44	0.4	0	0	0
'Nsg2'	0	0	0	0.81	0.63	0.44	0	0	0
'Slc25a54'	0	0	0.06	0.71	0.31	0.57	0.04	0	0
'LOC102557368'	0	0	0	0.58	0.32	0.35	0.05	0	0
'RT1-Ha'	0.13	0.1	0.09	1.2	0.65	0.26	0.14	0	0.07
'Lipg'	0	0.06	0	1.22	0.74	0.33	0	0	0
'Mcpt1'	0	0	0	0.89	0.71	0.21	0	0	0
'Il18r1'	0.04	0.07	0.15	1	0.95	0.47	0	0.07	0.07
'Mmp25'	0.06	0.12	0.13	0.85	1.22	0.35	0.25	0.12	0.06
'Il1rl1'	0.1	0.11	0.05	1.03	0.88	0.59	0.29	0.15	0.06
'Cxcr6'	0	0	0.2	1.75	0.8	1.39	0.1	0	0.09
'Sucnr1'	0	0	0	2.12	0.82	1.49	0	0	0
'Vax2'	0	0	0	2	1.03	0.71	0	0	0
'Apln'	0.05	0.16	0.11	1.85	1.33	0.6	0.03	0.11	0.1
'Cd80'	0.2	0.1	0.11	0.68	0.68	1.61	0.33	0.21	0.28
'Casz1'	0.13	0.15	0.15	0.78	0.8	0.92	0.17	0.17	0.16

'Galnt12'	0.11	0.08	0.12	0.62	0.98	1	0.12	0.06	0.26
'Gipc2'	0.23	0.15	0.08	0.61	0.98	0.93	0.24	0.08	0.35
'Asb11'	0	0	0	1.18	0.74	1.32	0	0	0
'Ptgs2'	0.1	0	0	1.19	0.73	1.28	0.21	0	0.09
'RGD1308065'	0	0	0	1.15	0.6	1.05	0.12	0	0
'Penk'	0	0	0	1.18	0.61	0.98	0.23	0.07	0.07
'RGD1562667'	0.06	0.06	0.06	1.14	0.58	0.87	0	0	0.21
'Strip2'	0.07	0.1	0.04	1.19	0.72	0.79	0.05	0.1	0.15
'F13a1'	0.1	0.05	0.03	1.22	0.64	0.67	0.15	0.05	0.15
'LOC691670'	0	0	0	1.04	0.48	0.89	0	0.16	0
'RT1-S2'	0.03	0.1	0.04	1.17	0.35	0.77	0.01	0.01	0.01
'Rab44'	0	0.02	0.02	0.62	0.98	0.96	0.23	0.07	0.07
'Il27'	0	0.09	0	0.76	0.98	0.93	0.09	0.04	0.12
'C1H19orf84'	0.12	0.12	0.02	0.73	0.93	0.88	0.15	0.07	0.04
'Slc35f2'	0	0.07	0.06	0.56	0.67	0.77	0.22	0.03	0.13
'Ankdd1a'	0	0	0	0.45	0.74	0.69	0.2	0.08	0.08
'Ffar2'	0	0	0	0.72	0.69	0.6	0.05	0.11	0.09
'Adgb'	0.02	0	0	0.77	0.89	0.65	0.16	0.06	0.16
'Izumo1r'	0	0	0	0.85	0.61	1.08	0.1	0	0
'Slc2a3'	0.04	0.09	0	0.71	0.54	1.36	0	0	0.09
'LOC102553150'	0	0	0.09	0.68	0.85	0.85	0	0.09	0
'Il18rap'	0.03	0	0.07	0.9	0.71	0.83	0.07	0	0.06
'Tnfsf8'	0.06	0.04	0.08	0.9	0.85	0.83	0.06	0	0
'Slain1'	0	0	0.04	1.03	0.79	0.98	0	0.12	0.04
'Ubash3a'	0.05	0.07	0.05	0.98	0.81	0.99	0.03	0.03	0.05
'Ly49i4'	0.14	0.21	0.1	0.85	0.71	0.81	0	0.07	0
'Trat1'	0.05	0.05	0.1	0.69	0.59	0.8	0	0.1	0.05
'Lrp8'	0.03	0.09	0.06	0.74	0.56	0.79	0.08	0.07	0.16
'Bpnt2'	0	3.25	0	0.01	0	0	0	3.55	0
'Gdf10'	0.56	0.47	0.79	0.24	0.11	0.11	0.58	0.51	0.65
'Slc29a4'	0.73	0.68	0.88	0.29	0.13	0.24	0.83	0.94	0.9
'Adgrg2'	0.82	0.58	1.06	0.24	0.08	0.19	0.83	0.78	0.62
'Hrc'	0.99	0.9	0.69	0.07	0.07	0.1	0.23	0.4	0.44
'Atp13a5'	1.1	0.9	1.14	0.04	0.05	0.09	0.38	0.29	0.24
'Smim35'	1.51	1.05	1.41	0.12	0.04	0.04	0.36	0.46	0.36
'Abcc8'	1.28	1.05	1.27	0.1	0.11	0.07	0.31	0.31	0.4
'Gstm6'	0.44	0.6	0.74	0.09	0.16	0.08	0.92	2.18	1.44
'Ucma'	1.18	0.98	1.23	0	0	0.32	1.4	1.33	1.21
'Ttll9'	0.77	1.18	0.92	0.12	0.14	0.1	1.64	1.61	1.31
'Polr2i'	1.32	1.31	2.09	0	0.61	0.21	1.37	1.52	0.81
'Fabp12'	2.41	1.09	1.09	0.02	0.17	0.15	0.84	0.73	1.13
'Spp1'	0.51	0.86	0.4	10.36	12.96	11.27	85.31	18.63	51.71
'LOC100911545'	4.72	4.15	4.95	31.94	30.74	22.07	134.96	94.49	105.01
'Ptprcap'	2.43	3.69	3.1	56.6	66.98	70.54	4.07	3.86	3.42

'Mx2'	3.02	4.93	4.11	75.3	69.58	71.18	3.58	2.29	3.15
'Slfn4'	5.91	6.47	5.76	88.99	69.49	80.43	4.17	3.84	2.87
'Fcgr3a'	3.99	4.23	3.55	95.36	85.94	74.59	5.02	3.69	3.09
'LOC100910270'	4.6	1.33	6.68	117.74	153.45	118.2	10.58	8.99	6.12
'Usp18'	5.06	4.95	5.17	64.25	44.58	60.26	7.48	8.45	8.21
'Ly86'	6.31	5.38	6.73	63.39	54.57	57.41	9.43	8.51	8.22
'Parp14'	5.45	5.62	5.73	66.37	47.69	60.7	10.69	9.2	8.48
'LOC691141'	4.1	2.87	4.13	67.03	64.86	68.05	10.81	7.37	8.09
'Bcl2a1'	2.66	4.67	4.08	64.51	66.13	59.87	12.74	8.4	9.43
'Isg15'	4.83	5.65	4.85	117.39	111.63	108.55	11.68	4.39	9.4
'RT1-N3'	9.09	7.56	8.7	92.31	102.65	104.56	10.51	10.22	10.57
'Coro1a'	7.46	7.05	5.96	89.78	113.98	106.89	10.94	8.51	11.95
'Irf7'	7.07	9.35	8.17	113.52	93.76	112.67	9.9	7.93	8.22
'Irf8'	6.99	6.75	6.38	100.47	103.48	108.63	8.87	7.26	6.68
'Ccl4'	4.1	5.93	3.81	53.45	42.57	46.48	3.95	4.5	6.56
'RT1-T24-3'	4.65	4.79	5.51	30.17	36.01	34.84	7.07	5.15	5.96
'Isg20'	5.54	5.27	5.8	33.79	41.76	28.39	7.74	5.61	7.51
'Ifi44'	4	5.19	4.02	33	24.24	27.17	4.92	5.87	3.99
'LOC108348241'	5.13	6.58	5.88	30.53	25	32.37	4.83	4.67	3.91
'Xaf1'	3.03	3.44	4.7	47.13	37.48	46.15	5	3.26	4.14
'Ifit1'	2.61	2.31	3.95	43.48	30.26	35.45	3.64	2.71	3.2
'Upp2'	5.29	4.54	5.35	30.02	25.97	26.88	3.24	2.24	2
'Rnf213'	3.48	3.43	3.81	24.73	19.82	22.95	3.99	3.71	3.32
'Arhgap9'	2.62	3.04	3.46	20.87	24.66	21.33	5.01	3.12	3.97
'Dhx58'	3.22	3.26	3.56	25.03	28.65	23.3	4.93	4.09	4.88
'Hk3'	1.32	1.29	1.23	58.35	69.71	60.29	4.4	3.05	3.43
'Cd8a'	2.06	1.81	1.58	66.29	76.91	71.36	4.56	3.6	2.94
'Neurl3'	2.95	3.02	2.28	75.53	71.46	86.08	8.08	3.4	5.94
'Tnfsf10'	2.38	3.64	2.84	87.57	73.91	79.7	5.48	4.41	4.64
'Il2rg'	2.34	2.06	1.65	57.43	57.4	66.48	11.71	7.45	7.71
'Bak1'	2.18	2.67	2.57	44.55	47.85	47.27	8.03	6.04	5.32
'Cd38'	3.79	3.74	3.93	51.57	41.61	40.99	10.09	8.71	6.36
'Nfam1'	3.93	3.6	3.04	45.37	44.55	45.6	10.49	7.51	7.22
'Lat'	1.75	1.23	1.92	26.76	32.9	31.38	3.73	4.95	7.14
'Evl'	2.96	2.46	2.67	23.85	35.87	31.4	4.78	3.97	4.62
'Ezr'	2.91	1.86	2.26	22.17	34.97	24.63	5.28	3.56	5.44
'Casp1'	3.26	4.06	2.48	35.52	31.15	30.8	6.46	5.05	6.2
'Pla2g2d'	2.76	3.77	2.83	35.22	32.07	34.87	8.08	6.81	5.55
'H3f3c'	2.24	1.75	2.15	42.2	27.77	39.9	7.13	4.8	4.81
'Tifa'	2.02	1.65	2.12	38.85	37.86	33.32	5.01	4.28	5.53
'Ripk2'	3.2	2.73	2.98	34.73	30.39	34.16	5.74	4.18	3.69
'Ms4a6a'	3.1	2.84	2.53	51.68	39.72	41.02	5.82	4.4	4.03
'Srgn'	8.56	12.15	11.01	70.12	72.15	71.04	13.24	8.05	10
'LOC103690108'	12.02	13.59	12.05	61.65	103.82	67.3	13.59	11.98	18.27

'Phf11'	13.54	10.69	10.85	77.96	63.24	76.48	14.68	13.5	13.74
'LOC100364500'	11.21	8.34	10.12	65.67	84.67	58.03	12.5	10.77	13.17
'Hcls1'	4.72	5.69	4.65	63.38	98.52	66.45	16.8	12.72	14.87
'Serpinb9'	7.13	9.14	9.39	93.03	79.81	81.07	18.93	15.56	14.26
'Slc11a1'	6.96	7.15	7.57	80.51	81.39	74.76	19.1	13.52	13.77
'Lcp1'	6.66	6.88	6.61	73.27	72.42	75.63	19.09	11.34	12.39
'Plvap'	7.35	6.11	7.4	41.15	73.6	47.05	10.09	7.94	9.25
'Lsp1'	8.6	8	7.84	54.57	79.9	65.97	10.49	6.08	10.37
'Oas1a'	6.41	5.95	6.24	60.37	65.94	66.94	9.1	6.87	8.41
'Rnf19b'	5.74	5.65	5.81	52.55	72.78	58.04	10.27	6.85	9.18
'Dtx3l'	11.2	8.33	7.78	60.41	52.97	42.26	13.3	13.73	9.76
'Trim5'	9.19	8.51	7.46	69.86	49.29	60.75	10.65	9.58	8.7
'RT1-Bb'	20.12	17.75	14.01	107.68	168.03	118.82	8.77	7.14	8.26
'Cxcl11'	13.87	15.56	16.25	136.42	103.88	119.82	12.28	13.9	10.73
'RT1-CE7'	13.67	13.95	15.21	88.76	133.81	106.26	11.98	8.36	13.15
'RT1-CE10'	17.24	16.68	16.28	98.22	161.4	112.22	13.11	11.89	17.39
'RT1-DMb'	11.55	13.35	11.22	129.68	111.02	118.18	23.67	22.36	21.57
'Apol9a'	16.19	17.28	16.27	109.99	117.65	102.03	18.73	17.4	19.25
'Ube2l6'	8.38	9.48	7.7	153.95	161.42	154.51	23.48	24.25	21.45
'Tap2'	9.31	9.15	8.98	146.12	165.01	172.35	14.21	11.89	12.3
'G6pc'	11.17	10.11	11	152.35	127.62	149.65	11.21	17.63	16.2
'Irgm'	3.14	2.94	2.58	370.53	282.56	307.27	24.99	22.94	23.78
'Apol3'	5.96	6.19	5.68	224.71	230.48	233.04	16.79	17.62	19.49
'Tap1'	6.02	6.33	5.89	212.13	207.16	231.54	13.49	10.82	11.16
'Upp1'	2.04	2.71	2.03	139.36	192.9	119.75	10.12	7.42	10.37
'Gbp5'	1.51	1.66	1.16	199.34	189.89	182.72	6.64	3.9	5.04
'LOC685067'	1.96	1.99	2.23	147.85	104.18	118.88	6.77	5.22	4.38
'LOC100911104'	2.11	2.06	2.78	309.83	287.65	375.9	6.45	4.51	3.98
'MGC108823'	5.3	3.34	2.92	511.19	347.39	488.26	11.61	9.58	8.08
'RGD1309362'	3.07	2.87	3.88	527.18	364.9	449.22	10.82	7.17	7.91
'LOC100910979'	3.1	2.93	4.21	360.24	265.83	271	13.79	7.21	7.76
'Gbp2'	3.55	4.26	3.19	330.1	321.21	310.54	9.57	5.59	5.32
'Acot1'	14.17	13.8	18.6	0.86	1.19	0.59	7.07	0.97	2.47
'Bhlha15'	10.52	9.94	11.11	0.85	1.3	1.67	6.45	5.08	5.98
'Adam11'	15.19	16.26	17.96	0.98	0.89	0.61	4.66	5.84	7.06
'Abcg5'	18.84	17.81	18.02	2.37	1.7	1.57	8.85	7.85	7.9
'Aldh1b1'	14.74	14.08	16.64	2.82	2.81	2.62	14.2	12	12.57
'Nrg4'	22.45	22.25	22.14	6.41	2.93	3.29	24.81	19.57	15.04
'Ddc'	37.1	32.99	33.13	4.25	4.29	4.23	17.23	16.32	17.02
'Tff3'	32.78	43.43	25.94	1.19	1.8	1.49	88.58	120.15	106.66
'Cyp3a23-3a1'	277.35	303.48	281.12	21.73	6.08	4.42	45.48	39.8	47.95
'Fgf21'	227.92	146.89	224.02	2.74	4.04	4.4	26.35	25.77	22.34
'Igfbp2'	86.1	89.49	81.7	10.8	13.25	15.92	110.24	70.06	99.77
'Acsm3'	102.42	89.6	99.18	13.15	10.51	9.81	58.94	55.94	46.36

'Hao2'	80.98	82.18	86.64	22.67	16.07	16.43	73.14	63.64	63.31
'Ugt2a3'	57.81	53.38	63.58	11.22	9.01	13.99	45.93	39.22	41.38
'Slc22a8'	54.1	53.82	58.32	7.43	2.53	6.48	49.74	47.56	40.92

Table S4. Ferroptosis-related genes

Gene Symbol	Ctrl Average TPM	LT Average TPM	HEXO Average TPM
'Gpx4'	409.73	297.3833	352.8367
'Gclm'	73.57333	26.58	37.21667
'Tfrc'	32.35333	11.15	15.70333
'Map1lc3b'	139.2133	117.3967	121.8867
'Steap3'	12.07667	5.476667	7.216667
'LOC100360087'	84.33333	41.18667	53.94667
'Ncoa4'	46.04667	26.46667	32.58667
'Slc39a8'	14.24333	11.27	9.346667
'Slc40a1'	25.37	18.38667	13.31333
'Sat2'	22.34667	15.33	13.93667
'Acsl1'	669.78	305.7333	302.9433
'Gclc'	194.5867	17.98333	29.17667
'Ftl1'	5718.593	3280.16	3429.483
'Acsl5'	83.75333	85.57	75.35667
'Slc3a2'	44.89	49.84667	43.35667
'Atg5'	10.82667	13.84	8.836667
'Vdac3'	77.43667	90.29	62.95
'Pcbp1'	157.1067	181.27	134.5267
'Vdac2'	64.90333	76.73667	54.67667
'Atg7'	3.943333	6.16	4.046667
'Sat1'	29.55333	85.66333	43.73333
'Hmox1'	53.74667	293.5667	119.4033
'Cybb'	3.226667	27.49667	14.62
'Tp53'	37.6	66.32667	49.73333
'Aifm2'	13.65	23.40667	19.75667
'Acsl4'	21.38667	46.21667	36.12
'Cp'	342.1033	2271.22	1700.75
'Pcbp2'	66.68333	81.07	77.35333
'Fth1'	2050.71	1996.563	2191.003
'Acsl3'	7.53	5.75	8.313333
'NEWGENE_620180'	27.18667	25.41333	28.01667
'Gss'	28.83333	30.31	40.17667
'Prnp'	2.063333	2.883333	6.47
'Slc7a11'	0.023333	0.033333	0.073333
'Alox15'	0.083333	1.453333	4.106667
'Tf'	19147.09	21029.92	26328.94
'Slc11a2'	10.04	14.00667	15.97667

'Lpcat3'	44.75	60.70333	62.03333
'Slc39a14'	49.25333	85.92	82.75667
'Map1lc3a'	66.15667	92.54333	92.39
